# Mesoscopic population equations for spiking neural networks with synaptic short-term plasticity

**DOI:** 10.1186/s13408-020-00082-z

**Published:** 2020-04-06

**Authors:** Valentin Schmutz, Wulfram Gerstner, Tilo Schwalger

**Affiliations:** 1 Brain Mind Institute, École Polytechnique Féderale de Lausanne (EPFL), Lausanne, Switzerland; 2 Bernstein Center for Computational Neuroscience, Institut für Mathematik, Technische Universität Berlin, Berlin, Germany

**Keywords:** Short-term plasticity, Multi-scale modeling, Mesoscopic population dynamics

## Abstract

Coarse-graining microscopic models of biological neural networks to obtain mesoscopic models of neural activities is an essential step towards multi-scale models of the brain. Here, we extend a recent theory for mesoscopic population dynamics with *static* synapses to the case of *dynamic* synapses exhibiting short-term plasticity (STP). The extended theory offers an approximate mean-field dynamics for the synaptic input currents arising from populations of spiking neurons and synapses undergoing Tsodyks–Markram STP. The approximate mean-field dynamics accounts for both finite number of synapses and correlation between the two synaptic variables of the model (utilization and available resources) and its numerical implementation is simple. Comparisons with Monte Carlo simulations of the microscopic model show that in both feedforward and recurrent networks, the mesoscopic mean-field model accurately reproduces the first- and second-order statistics of the total synaptic input into a postsynaptic neuron and accounts for stochastic switches between Up and Down states and for population spikes. The extended mesoscopic population theory of spiking neural networks with STP may be useful for a systematic reduction of detailed biophysical models of cortical microcircuits to numerically efficient and mathematically tractable mean-field models.

## Introduction

One of the primary goals in computational neuroscience is to understand how brain functions arise from the interactions of billions of nerve cells and their underlying biophysical processes at the microscopic scale. Towards that goal, a crucial step is to develop a theoretical framework that links biophysically detailed networks of spiking neurons at the microscopic scale with simplified firing-rate or neural-mass models [1] for neuronal populations at the coarse-grained mesoscopic or macroscopic scale. Firing-rate models are mathematically tractable and thus permit a theoretical understanding of neuronal population dynamics implicated in various neural computations [2–6]. However, firing rate models are heuristic models that lack a clear link to the underlying microscopic properties. On the other hand, highly detailed biophysical models of cortical microcircuits [7] and simplified networks of spiking point neurons [8–10] are closely linked to biophysical properties but lack mathematical tractability and do not provide a mechanistic understanding of emergent functional behavior. However, if we were able to systematically reduce biophysically detailed models to simplified networks of spiking point neurons [11] and further to coarse-grained firing-rate models [12], we might be able to understand neural computations at the population level in terms of biophysical parameters. One common strategy for coarse-graining a cortical network is to identify approximately homogeneous neuronal populations that consist of cells with similar properties and inputs; e.g., neurons may be grouped with respect to their cell type and cortical location [12, 13]. Biological data suggests that the size of such neuronal populations are typically small containing only on the order of hundred to thousand of neurons [14]. Recently, a *mesoscopic* mean-field theory that accounts for finite-size noise has been proposed [12] based on the refractory density equations for *macroscopic* homogeneous populations of neurons (where the number of neurons tends to infinity) [15–18]. However, in [12], synapses are assumed to be *static* in the sense that the effective synaptic coupling between two populations of neurons is constant over time.

A ubiquitous feature of cortical dynamics is synaptic short-term plasticity (STP) [19–22], i.e. dynamic changes of synaptic strength on time scales of 100 ms to 1000 ms induced by presynaptic neural activity. Theoretical studies have shown that STP exerts profound effects on network activity [23–25] and information processing capabilities [19, 26–29]. In particular, in a recent biophysically detailed microcircuit model [7], STP has been a critical factor for reproducing experimentally observed activity patterns. Therefore, a faithful reduction to population rate models should incorporate the effect of STP. Mean-field descriptions for populations of spiking neurons with dynamic synapses are central for such a reduction. Although mean-field theories for STP have been developed for the case of *macroscopic* populations [4, 30–32], a *mesoscopic* mean-field theory that would account for finite-size fluctuations is still lacking.

In this work, we extend the mesoscopic theory of [12] for *static* synapses to the case of *dynamic* synapses exhibiting Tsodyks–Markram STP [30]. In Sect. 2 we expose our theory by considering a feedforward setup [27, 33]. We use an assumption (loosely speaking a Poissonian assumption on the spike statistics), Assumption 1, which enables the derivation of mesoscopic mean-field dynamics for the effective input. We then compare numerically the effective input given by the mesoscopic mean-field dynamics with simulations of the full microscopic population, in the case where the presynaptic population consists of *N* Poisson neurons. The Poisson case is of special interest because Assumption 1 is satisfied. In Sect. 3, we first explain how the theory of Sect. 2 can be applied to general mesoscopic circuits. We then illustrate how the mesoscopic STP model accurately replicates population spikes and switches between Up and Down states exhibited by a recurrent network of time-inhomogeneous Poisson neurons. Finally, we incorporate the mesoscopic STP model into our previous mesoscopic population model [12] for generalized integrate and fire (GIF) neurons. We show that the resulting extension faithfully reproduces population spikes observed in a microscopic simulation. In Sect. 4, we discuss the limitations of our mesoscopic model for GIF neurons and mention possible theoretical extensions. The numerical implementation of the mesoscopic equations are detailed in the Appendix.

## Feedforward network with a finite number of dynamic synapses

### Network setting and theoretical approach

**Figure 1:**
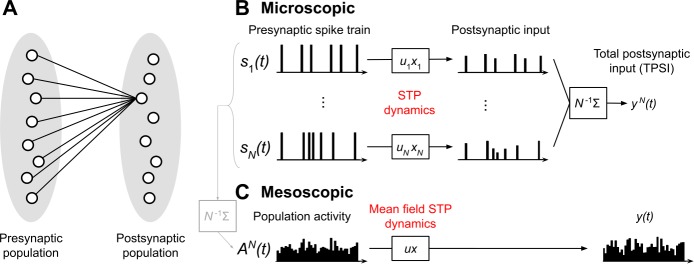
*Illustration of the setup in the feedforward network*. (**A**) Two populations connected in a feedforward manner via dynamic synapses. We focus on the connections from neurons *j*, $j=1,\ldots ,N$, in the presynaptic population to a specific postsynaptic neuron *i*. (**B**) Microscopic picture of *N* presynaptic spike trains $s_{j}(t)$ driving the STP dynamics of $u_{j}(t)$ and $x_{j}(t)$ for each of the *N* synapses. The postsynaptic input resulting from synapse *j* is $u_{j}(t)x_{j}(t)s_{j}(t)$ and the total postsynaptic input is $y(t)=N^{-1}\sum_{j=1}^{N}u_{j}(t)x_{j}(t)s_{j}(t)$. (**C**) Mesoscopic picture of *one* effective synapse with *mean-field* STP dynamics driven by the population activity $A^{N}(t)$ of *N* neurons. The population activity $A^{N}(t)$ is defined as the population average of the spike trains of each of the *N* neurons forming the population. Thus, when the individual spike trains $s_{j}(t)$ are known, $A^{N}(t)=N^{-1}\sum_{j=1}^{N} s_{j}(t)$

To derive the mesoscopic theory, let us consider a feedforward setup: *N* neurons from a presynaptic population are connected to a given postsynaptic neuron via *N* synapses (Fig. 1(A)). This setup is important because under the approximations we will use, the theory obtained for this simple case can be directly applied to general mesoscopic circuits. In addition, the setup is important for biological modeling because feedforward pathways exhibiting STP are prominent in the nervous system. Examples include visual [19], auditory [34], somatosensory [35] and periform [36] cortices.

Before we start with the treatment of dynamic synapses, it is instructive to first recall the simpler case of static synapses [12]. In this case, the synaptic input current (in a current-based model) $I_{\mathrm{syn}}^{N}(t)$, $t>t_{0}$, is modeled microscopically as the sum of synaptically-filtered spike trains:

1 $$ I_{\mathrm{syn}}^{N}(t)=\sum _{j=1}^{N}\frac{J}{N} \int _{t_{0}}^{t} \epsilon \bigl(t-t' \bigr)s_{j}\bigl(t'\bigr) \,dt'. $$

Here, *J* is the synaptic weight (in units of electrical charge) assumed to be identical for all synapses onto the postsynaptic neuron, $\epsilon (t)$ is a synaptic filtering kernel (defined as the postsynaptic current normalized by the total charge elicited by a single spike) and

2 $$ s_{j}(t)=\sum_{k\in \mathbb{Z}^{+}} \delta \bigl(t-t_{k}^{j}\bigr) $$

is the Dirac-delta spike train of neuron *j* with spike times $\{t_{k}^{j}\}_{k\in \mathbb{Z}^{+}}$, $t_{0}< t_{1}^{j}< t_{2}^{j}<\cdots <\infty $. Here and in the following, a superscript *N* (as in $I_{\mathrm{syn}}^{N}$) denotes a functional of the *N* presynaptic spike trains $s_{1}(t), \ldots , s_{N}(t)$, and hence a mesoscopic quantity.

In mesoscopic models of homogeneous neuronal populations, the central mesoscopic variable is the population activity $A^{N}(t)$ defined as the sum of spike trains of all neurons in a population divided by the number of neurons. In our case, the mesoscopic activity of the presynaptic population is thus given by

3 $$ A^{N}(t)=\frac{1}{N}\sum _{j=1}^{N}s_{j}(t). $$

Using the mesoscopic activity of the presynaptic population, the synaptic input current, Eq. (1), can be rewritten as

4 $$ I_{\mathrm{syn}}^{N}(t)=J \int _{t_{0}}^{t}\epsilon \bigl(t-t' \bigr)A^{N}\bigl(t'\bigr) \,dt'. $$

Thus, for static synapses, the synaptic input current is completely determined by the past population activities $\{A^{N}(t')\}_{t'< t}$. This property is crucial for mesoscopic population models that are formulated in terms of mesoscopic population activities [12, 37]. In particular, in simulations of such mesoscopic models forward in time, the information about past population activities is available at each point in time and can thus be used to compute the input current at present time. In our approach, we thus aim at finding a dynamics of the synaptic input *conditioned* on the history of the population activity $A^{N}(t)$. We would like to stress that this aim is markedly different from well-known diffusion approximations [38, 39] (see also [40, 41] for examples in neuroscience), where a jump process is approximated, when jumps become frequent and small, by a diffusion process. In particular, a diffusion approximation would yield a *stochastic* dynamics if conditioned on the past population activities, in stark contrast to the *deterministic* conditional dynamics, Eq. (4).

Finding a deterministic relationship between the synaptic input and a given realization $A^{N}(t)$, as in Eq. (4), is no longer possible in the case of dynamic synapses. In this case, the input spike trains $s_{j}(t)$ are modulated by a dynamic factor $R_{j}(t)$ modeling the effect of STP. In contrast to static synapses, Eq. (1), the synaptic current for dynamic synapses reads

5 $$ I_{\mathrm{syn}}^{N}(t)=\sum_{j=1}^{N} \frac{J}{N} \int _{t_{0}}^{t} \epsilon \bigl(t-t' \bigr)R_{j}\bigl(t'\bigr)s_{j} \bigl(t'\bigr) \,dt'. $$

Inverting the sum and the integral we get

6 $$ I_{\mathrm{syn}}^{N}(t) = J \int _{t_{0}}^{t}\epsilon \bigl(t-t' \bigr)y^{N}\bigl(t'\bigr) \,dt',\qquad y^{N}(t)= \frac{1}{N}\sum_{j=1}^{N} R_{j}(t)s_{j}(t), $$

where we introduced the *total postsynaptic input* (TPSI) $y^{N}(t)$. Equation (6) shows that determining the synaptic input $I_{\mathrm{syn}}^{N}(t)$ from the knowledge of the population activity $A^{N}(t)$ is an underconstrained problem because $y^{N}(t)$ is a weighted average of spike trains whereas $A^{N}(t)$ is an unweighted average. Thus, in mesoscopic simulations, we expect that the synaptic input is strongly but not fully constrained by the knowledge of past population activities. To capture STP in a mesoscopic model, our approach is to find an approximate mean-field dynamics for $y^{N}(t)$ by introducing additional mesoscopic variables that are driven by the population activity and noise that accounts for the inevitable uncertainty of $y^{N}(t)$ given the history of the population activity. Importantly, such mean-field dynamics would be mesoscopic in the sense that its simulation does not require simulating all the individual presynaptic neurons and synapses but only a few mesoscopic variables. In addition, for such an approximation to be useful for coarse-graining, its numerical implementation should be computationally economical compared with the simulation of the full microscopic model.

Now we fully specify the dynamics of $y^{N}(t)$ as defined Eq. (6) (the microscopic model) in Sect. 2.2 and derive its mesoscopic approximation in Sect. 2.3.

### Microscopic model

In order to fully specify the dynamics of $y^{N}(t)$ defined in Eq. (6) given the collection of spike trains $\{s_{j}\}_{j=1, \ldots , N}$, we need to define the dynamics of $\{R_{j}\}_{j=1, \ldots , N}$, which modulates the amplitude of the spikes in $s_{j}(t)$. For each synapse *j*, the time evolution of $R_{j}$ is deterministic given $s_{j}$ and follows the Tsodyks–Markram model for STP [30]. The modulation factor $R_{j}(t)$ can be seen as the amount of neurotransmitter that would be released if a spike occurs at time *t*. It is given by the product of two synaptic variables, $R_{j}(t)=u_{j}(t^{-}) x_{j}(t^{-})$, where $x_{j}(t)$ is the amount of available synaptic resources and $u_{j}(t)$ is the utilization of available resources (i.e. the fraction of these available resources that would be released if a spike occurs) at synapse *j*. Given the presynaptic spike trains $s_{j}(t)$, these variables obey the dynamics

7a $$\begin{aligned}& \frac{\mathrm{d}u_{j}(t)}{\mathrm{d}t} = \frac{U_{0}-u_{j}(t)}{\tau _{F}}+U \bigl(1-u_{j}\bigl(t^{-}\bigr)\bigr)s_{j}(t), \end{aligned}$$

7b $$\begin{aligned}& \frac{\mathrm{d}x_{j}(t)}{\mathrm{d}t} = \frac{1-x_{j}(t)}{\tau _{D}}-u_{j} \bigl(t^{-}\bigr)x_{j}\bigl(t^{-} \bigr)s_{j}(t), \end{aligned}$$

where $\tau _{\mathrm {F}}$ and $\tau _{\mathrm {D}}$ are the facilitation and depression time constants, respectively, $U_{0}$ is the baseline utilization of synaptic resources, *U* determines the increase in the utilization of synaptic resources by a spike. Here, $u_{j}(t^{-})$ is a shorthand for the left limit at time *t*. Note that the presence of the product $u_{j} x_{j}$ in Eq. (7b) introduces a nonlinear coupling between the dynamics of $u_{j}$ and $x_{j}$. Having specified the STP dynamics, we can rewrite the TPSI, Eq. (6), as follows:

8 $$ y^{N}(t) = \frac{1}{N}\sum _{j=1}^{N} u_{j}\bigl(t^{-} \bigr) x_{j}\bigl(t^{-}\bigr) s_{j}(t). $$

In the next section, we present our main result, which provides a mean-field approximation $y(t)$ that is determined by the history of the mesoscopic presynaptic population activity $A^{N}(t)$ rather than individual presynaptic spike trains $s_{j}(t)$ or synaptic variables $u_{j}$ and $x_{j}$.

### Mesoscopic approximation

To relate the TPSI, Eq. (8), to the presynaptic population activity we consider the collection $\{t_{k}\}_{k\in \mathbb{Z}^{+}}$ of all spike times of the superposition spike train $S^{N}(t):=NA^{N}(t)=\sum_{j} s_{j}$. At the mesoscopic scale, we only know that there is a presynaptic spike arriving at time $t_{k}$ but we do not know at which synapse. That is, we do not know the mapping $j(k)$ that maps spike arrival times $t_{k}$ to specific synapses *j*. In terms of the spike times $t_{k}$ and the mapping $j(k)$, the population activity and the TPSI can be rewritten as

9 $$ A^{N}(t)=\frac{1}{N}\sum _{k\in \mathbb{Z}^{+}}\delta (t-t_{k}) $$

and

10 $$ y^{N}(t)=\frac{1}{N}\sum _{k\in \mathbb{Z}^{+}}u_{j(k)}\bigl(t^{-} \bigr)x_{j(k)}\bigl(t^{-}\bigr) \delta (t-t_{k}), $$

respectively. Equation (10) still contains the microscopic variables $u_{j}$ and $x_{j}$ and thus the precise spike arrival times $t_{k}^{j}$ at synapse *j*. To derive a mean-field approximation $y(t)$ that only depends on the spike times $\{t_{k}\}_{k\in \mathbb{Z}_{+}}$ of the superposition spike train $S^{N}(t)$, we make two approximation steps: (i) a randomization of synapse indices $j(k)$ at each spike time $t_{k}$ and (ii) a Gaussian approximation concerning the variables $u_{j(k)}(t)$ and $x_{j(k)}(t)$ determining the “amplitudes” of the delta spikes in Eq. (10). The purpose of the first approximation step is a probabilistic description of the values $u_{j(k)}(t_{k}^{-})$ and $x_{j(k)}(t_{k}^{-})$ upon spiking that accounts for the lack of knowledge about synapse identities $j(k)$ at spike times $t_{k}$. This step rests on an assumption on the law of $\{s_{j}\}_{j=1, \ldots , N}$. In feedforward models with biologically realistic spike train statistics or in recurrent networks of spiking neurons, this assumption has to be understood as a useful approximation as it is not satisfied in general.

#### Assumption 1

The set of spike trains $\{s_{j}\}_{j=1, \ldots , N}$ has the same law as a set of spike trains $\{s^{*}_{j^{*}}\}_{j^{*}=1, \ldots , N}$ that is constructed by picking independently and uniformly, for each spike time $\{t_{k}\}_{k \in \mathbb{Z}^{+}}$ of the superposition spike train $S^{N}(t)$, an integer $j^{*}(k)$ between 1 and *N* and assigning a spike to neuron $j^{*}(k)$ at time $t_{k}$.

An important case for which Assumption 1 is satisfied is the case where $\{s_{j}\}_{j=1, \ldots , N}$ is given by *N* independent Poisson processes with the same (time-varying) intensity. This special case will be extensively used in numerical simulations. Note that Assumption 1 is strictly weaker than assuming that the spike trains of the neurons are given by independent Poisson processes. Here is a simple counterexample: consider the process where at each time $t_{k}=t_{0}+k\hat{\tau }$ for $k \in \mathbb{Z}_{+}$ and $\hat{\tau }>0$, a spike occurs at neuron $j_{k}$, $j_{k}$ being chosen randomly, uniformly and independently at each time $t_{k}$. This process satisfies Assumption 1 but is clearly not Poisson.

Assumption 1 will be useful in the following because it will enable us to replace the unknown synapse $j(k)$ at some spike time $t_{k}$ by a randomly chosen synapse $j^{*}(k)$. This randomization of synapse indices yields a new set of presynaptic spike trains $\{s^{*}_{j}\}_{j=1, \ldots , N}$ whose population activity $A^{*}(t):=\frac{1}{N}\sum_{j=1}^{N}s_{j}^{*}(t)$ is identical to the population activity $A^{N}(t)$ and which by assumption is statistically equivalent to the original spike trains $\{s_{j}\}_{j=1, \ldots , N}$. Furthermore, the spike trains $\{s^{*}_{j}\}_{j=1, \ldots , N}$ induce new STP variables $u_{j}^{*}$ and $x_{j}^{*}$ that obey, analog to Eqs. (7a)–(7b), the dynamics

11a $$\begin{aligned}& \frac{\mathrm{d}u_{j}^{*}(t)}{\mathrm{d}t} = \frac{U_{0}-u_{j}^{*}(t)}{\tau _{F}}+U \bigl(1-u_{j}^{*}\bigl(t^{-}\bigr) \bigr)s_{j}^{*}(t), \end{aligned}$$

11b $$\begin{aligned}& \frac{\mathrm{d}x_{j}^{*}(t)}{\mathrm{d}t} = \frac{1-x_{j}^{*}(t)}{\tau _{D}}-u_{j}^{*} \bigl(t^{-}\bigr)x_{j}^{*} \bigl(t^{-}\bigr)s_{j}^{*}(t) , \end{aligned}$$

with initial conditions $u_{j}^{*}(t_{0})=u_{j}(t_{0})$, $x_{j}^{*}(t_{0})=x_{j}(t_{0})$. Assumption 1 implies that the jump processes $[u_{j}^{*}(t),x_{j}^{*}(t)]$, $j=1,\ldots ,N$, have the same law as the original STP variables $[u_{j}(t),x_{j}(t)]$, $j=1,\ldots ,N$.

Using the random indices $j^{*}(k)$, we define a two-dimensional càdlàg jump process $\{(\hat{u}^{*}(t), \hat{x}^{*}(t))\}_{t >t_{0}}$: For all $k \in \mathbb{Z}^{+}$, if $t \in [t_{k}, t_{k+1}[$ we set

12 $$ \hat{u}^{*}(t):=u_{j^{*}(k)}^{*}(t),\qquad \hat{x}^{*}(t) :=x_{j^{*}(k)}^{*}(t), $$

i.e. $\hat{u}^{*}(t)$ and $\hat{u}^{*}(t)$ are equal to $u_{j}^{*}(t)$ and $x_{j}^{*}(t)$ of that neuron *j* which had the most recent spike prior or equal to time *t*. In the following, the process $\{(\hat{u}^{*}(t), \hat{x}^{*}(t))\}_{t>t_{0}}$ will appear in the STP dynamics only as prefactors of Dirac-delta spikes. Therefore, only the values $(\hat{u}^{*}(t_{k}), \hat{x}^{*}(t_{k}))$ at times $t_{k}$ will enter the dynamics. The formulation at all time $t>t_{0}$, Eq. (12), will allow us to factorize modulated spike trains into modulation factor and population activity. For example, using the process $(\hat{u}^{*}(t), \hat{x}^{*}(t))$ we can define the new quantity

13 $$\begin{aligned} y^{*}(t)&:={\frac{1}{N}\sum _{k \in \mathbb{Z}^{+}}\hat{u}^{*}(t_{k}) \hat{x}^{*}(t_{k})\delta (t-t_{k})} = \frac{1}{N}\sum_{k \in \mathbb{Z}^{+}}\hat{u}^{*}(t) \hat{x}^{*}(t)\delta (t-t_{k}) \end{aligned}$$

14 $$\begin{aligned} &=\hat{u}^{*}(t)\hat{x}^{*}(t)A^{N}(t)= \hat{u}^{*}\hat{x}^{*}A^{N}(t) , \end{aligned}$$

where the second equality in Eq. (13) follows from the properties of the Dirac-delta function. In the last equation, we have introduced the shorthand notation $\hat{u}^{*}:=\hat{u}^{*}(t)$ and $\hat{x}^{*}:=\hat{x}^{*}(t)$. By construction of the processes $\hat{u}^{*}(t)$ and $\hat{x}^{*}(t)$, we can state an important fact.

#### Fact 1

*Under Assumption *1, *the processes*$y^{N}(t)$*and*$y^{*}(t)$*have the same law*. *Moreover*, *conditioned on the population activity*$A^{N}(t)$, *both processes have the same spike times*.

Therefore, we expect that $y^{*}(t)$ yields an accurate approximation of the TPSI $y^{N}(t)$ that is not only statistically equivalent but also captures the precise realization of spike times of the mesoscopic activity $A^{N}(t)$. However, $y^{*}(t)$ is not yet useful for a mesoscopic simulation because the process $(\hat{u}^{*}(t), \hat{x}^{*}(t))$ is constructed from the microscopic variables $u_{j}^{*}(t)$ and $x_{j}^{*}(t)$, $j=1,\ldots ,N$, which still need to be simulated individually.

In order to obtain an approximation that does not rely on microscopic simulations, we introduce new mesoscopic variables given by the empirical means and covariances of $u_{j}(t)$ and $x_{j}(t)$:

15a $$\begin{aligned}& u^{N}(t):=\frac{1}{N}\sum_{j=1}^{N}u_{j}(t),\qquad x^{N}(t):= \frac{1}{N}\sum_{j=1}^{N}x_{j}(t), \end{aligned}$$

15b $$\begin{aligned}& P^{N}(t):=\frac{1}{N}\sum _{j=1}^{N}u_{j}^{2}(t),\qquad Q^{N}(t):= \frac{1}{N}\sum _{j=1}^{N}x_{j}^{2}(t), \end{aligned}$$

15c $$\begin{aligned}& R^{N}(t):=\frac{1}{N}\sum _{j=1}^{N}u_{j}(t)x_{j}(t). \end{aligned}$$

Similar to $y^{N}$, we can find an accurate approximation for the new mesoscopic variables using analog definitions based on the asterisk (^∗^) variables:

16a $$\begin{aligned}& u^{*}(t):=\frac{1}{N}\sum _{j=1}^{N}u_{j}^{*}(t),\qquad x^{*}(t):= \frac{1}{N}\sum_{j=1}^{N}x_{j}^{*}(t), \end{aligned}$$

16b $$\begin{aligned}& P^{*}(t):=\frac{1}{N}\sum _{j=1}^{N}u_{j}^{*2}(t),\qquad Q^{*}(t):= \frac{1}{N}\sum_{j=1}^{N}x_{j}^{*2}(t), \end{aligned}$$

16c $$\begin{aligned}& R^{*}(t):=\frac{1}{N}\sum _{j=1}^{N}u_{j}^{*}(t)x_{j}^{*}(t). \end{aligned}$$

Because $[u_{j},x_{j}]$ and $[u_{j}^{*},x_{j}^{*}]$ have the same law, we can state the following.

#### Fact 2

*The processes*$[u^{*}, x^{*}, P^{*}, Q^{*}, R^{*}]$*and*$[u^{N},x^{N},P^{N},Q^{N},R^{N}]$*are multi*-*dimensional jump processes that have the same law*. *Moreover*, *conditioned on the population activity*$A^{N}(t)$, *the two jump processes have the same jump times*.

#### Lemma 2.1

*The jump process*$[u^{*}, x^{*}, P^{*}, Q^{*}, R^{*}]$*obeys the dynamics *

17a $$\begin{aligned}& \frac{\mathrm{d}u^{*}}{\mathrm{d}t} = \frac{U_{0}-u^{*}}{\tau _{F}}+U\bigl(1- \hat{u}^{*}\bigr)A^{N}(t), \end{aligned}$$

17b $$\begin{aligned}& \frac{\mathrm{d}x^{*}}{\mathrm{d}t} =\frac{1-x^{*}}{\tau _{D}}- \hat{u}^{*}\hat{x}^{*}A^{N}(t), \end{aligned}$$

17c $$\begin{aligned}& \frac{\mathrm{d}P^{*}}{\mathrm{d}t} =2 \frac{U_{0}u^{*}-P^{*}}{\tau _{F}}+U\bigl(1- \hat{u}^{*}\bigr)\bigl[(2-U) \hat{u}^{*}+U \bigr]A^{N}(t), \end{aligned}$$

17d $$\begin{aligned}& \frac{\mathrm{d}Q^{*}}{\mathrm{d}t} =2 \frac{x^{*}-Q^{*}}{\tau _{D}}- \hat{u}^{*}\hat{x}^{*2}\bigl(2- \hat{u}^{*} \bigr)A^{N}(t), \end{aligned}$$

17e $$\begin{aligned}& \frac{\mathrm{d}R^{*}}{\mathrm{d}t} = \frac{U_{0}x^{*}-R^{*}}{\tau _{F}}+ \frac{u^{*}-R^{*}}{\tau _{D}}+\hat{x}^{*} \bigl[U\bigl(1-\hat{u}^{*} \bigr)^{2}- \hat{u}^{*2} \bigr]A^{N}(t), \end{aligned}$$

*with initial conditions*$u^{*}(t_{0})=u^{N}(t_{0})$, $x^{*}(t_{0})=x^{N}(t_{0})$, $P^{*}(t_{0})=P^{N}(t_{0})$, $Q^{*}(t_{0})=Q^{N}(t_{0})$, $R^{*}(t_{0})=R^{N}(t_{0})$.

The proof is presented in Appendix A.

So far, we obtained approximations $y^{*}$ and $[u^{*},x^{*},P^{*},Q^{*},R^{*}]$, in which $\hat{u}^{*}$ and $\hat{x}^{*}$ are chosen randomly at spike times so as to account for the missing information about synapse identities at the microscopic scale. However, the process $[u^{*}(t),x^{*}(t),P^{*}(t),Q^{*}(t),R^{*}(t)]$ is not yet useful for a mesoscopic simulation because the processes $\hat{u}^{*}(t)$ and $\hat{x}^{*}(t)$ require the simulation of the microscopic variables $u_{j}^{*}(t)$ and $x_{j}^{*}(t)$, $j=1,\ldots ,N$. To resolve this problem, we make a Gaussian approximation of the two-dimensional random variables $(\hat{u}^{*}(t_{k}), \hat{x}^{*}(t_{k}))$ that is solely based on mesoscopic variables. We note again that only the values $(\hat{u}^{*}(t_{k}), \hat{x}^{*}(t_{k}))$ at spike times matter for the STP dynamics marked with asterisks, Eqs. (14) and (17a)–(17e), because they only appear as prefactors of Dirac delta spikes. For a Gaussian approximation of $[\hat{u}^{*}(t_{k}), \hat{x}^{*}(t_{k})]$, we need its first two empirical moments given by Eqs. (16a)–(16c). This means that at each spike $t_{k}$ of $A^{N}(t)$, a sample $[\hat{u}(t_{k}), \hat{x}(t_{k})]$ is drawn from a bivariate Gaussian distribution parametrized by $[u^{*}(t_{k}^{-}),x^{*}(t_{k}^{-}),P^{*}(t_{k}^{-}),Q^{*}(t_{k}^{-}),R^{*}(t_{k}^{-})]$. To close the system, we also need to approximate $[u^{*},x^{*},P^{*},Q^{*},R^{*}]$ by new variables $[u,x,P,Q,R]$ that obey Eqs. (17a)–(17e) but in which $[\hat{u}^{*}(t_{k}), \hat{x}^{*}(t_{k})]$ is replaced by its Gaussian approximation $[\hat{u}(t_{k}), \hat{x}(t_{k})]$. Likewise, we use $[\hat{u}(t_{k}), \hat{x}(t_{k})]$ in Eq. (14) to approximate the TPSI $y^{*}$ by a variable *y*. Thus, we obtain our main result.

#### Second-order mean-field approximation

*Let*$\{t_{k}\}_{k \in \mathbb{Z}^{+}}$*be the spike times of superposition spike train*$S=NA^{N}$. *Consider the process*$(u(t), x(t), P(t), Q(t), R(t), \hat{u}(t), \hat{x}(t))$*defined by the system of equations *

18a $$\begin{aligned}& \frac{\mathrm{d}u}{\mathrm{d}t} =\frac{U_{0}-u}{\tau _{F}}+U(1- \hat{u})A^{N}(t), \end{aligned}$$

18b $$\begin{aligned}& \frac{\mathrm{d}x}{\mathrm{d}t} =\frac{1-x}{\tau _{D}}-\hat{u} \hat{x}A^{N}(t), \end{aligned}$$

18c $$\begin{aligned}& \frac{\mathrm{d}P}{\mathrm{d}t} =2 \frac{U_{0}u-P}{\tau _{F}}+U(1-\hat{u}) \bigl[(2-U)\hat{u}+U\bigr]A^{N}(t), \end{aligned}$$

18d $$\begin{aligned}& \frac{\mathrm{d}Q}{\mathrm{d}t} =2\frac{x-Q}{\tau _{D}}- \hat{u} \hat{x}^{2}(2-\hat{u})A^{N}(t), \end{aligned}$$

18e $$\begin{aligned}& \frac{\mathrm{d}R}{\mathrm{d}t} = \frac{U_{0}x-R}{\tau _{F}}+ \frac{u-R}{\tau _{D}}+\hat{x} \bigl[U(1-\hat{u})^{2}- \hat{u}^{2} \bigr]A^{N}(t), \end{aligned}$$

*where at each spike time*$\{t_{k}\}_{k \in \mathbb{Z}^{+}}$, *a random sample*$[\hat{u}(t_{k}), \hat{x}(t_{k})]$*is drawn from the two*-*dimensional Gaussian distribution parametrized by*$[u,x,P,Q,R]$:

18f $$ \begin{pmatrix} \hat{u}(t_{k}) \\ \hat{x}(t_{k}) \end{pmatrix} \sim \mathcal{N} \left( \begin{pmatrix} u(t_{k}^{-}) \\ x(t_{k}^{-}) \end{pmatrix} , \begin{pmatrix} P(t_{k}^{-}) - u(t_{k}^{-})^{2} & R(t_{k}^{-}) - u(t_{k}^{-}){x(t_{k}^{-})} \\ R(t_{k}^{-}) - u(t_{k}^{-})x(t_{k}^{-}) & Q(t_{k}^{-}) - x(t_{k}^{-})^{2} \end{pmatrix} \right) , $$

*and for the irrelevant values between spikes we arbitrarily set*$(\hat{u}(t), \hat{x}(t)) = (\hat{u}(t_{k}), \hat{x}(t_{k}))$*if*$t \in [t_{k}, t_{k+1}[$*for all*$k \in \mathbb{Z}^{+}$. *The mesoscopic mean*-*field approximation of the synaptic input is*

18g $$ \begin{aligned} &y^{N}(t) \approx y(t):= \hat{u}(t) \hat{x}(t)A^{N}(t){=\frac{1}{N} \sum _{k}\hat{u}(t_{k})\hat{x}(t_{k}) \delta (t-t_{k})}, \\ &I_{\mathrm{syn}}^{N}(t) \approx I_{\mathrm{syn}}(t):= J \int _{t_{0}}^{t}\epsilon \bigl(t-t' \bigr)y\bigl(t'\bigr) \,dt'. \end{aligned} $$

#### Remarks on the approximation

1. Various initial conditions are possible for the mesoscopic dynamics. One reasonable choice is $u(t_{0})=0$, $x(t_{0})=1$, $P(t_{0})=Q(t_{0})=R(t_{0})=0$.
2. The approximation is mesoscopic because the process $(u, x, P, Q, R, \hat{u}, \hat{x})$ defined by Eqs. (18a)–(18e) and (18f) does not involve any microscopic simulations. The process is solely driven by the mesoscopic population activity $A^{N}(t)$. The key heuristic we use is the Gaussian approximation of the random variables $u_{j^{*}(k)}(t_{k}^{-})$ and $x_{j^{*}(k)}(t_{k}^{-})$ at spike times $t_{k}$.
3. In principle, the jump process, Eqs. (18a)–(18g), can be simulated by a discrete-time forward scheme, where at each spike time $t_{k}$ a bivariate Gaussian random number is drawn. In practice, however, such a simulation would be inefficient because for large *N* the rate of spike times $t_{k}$ is large and thus the discretization time step needs to be chosen extremely small in order to resolve each spike time. In Appendix B, we present an efficient simulation scheme that allows many spikes per time step.
4. The process $(u, x, P, Q, R, \hat{u}, \hat{x})$ associated with the mesoscopic approximation is well defined. First, as the number of neurons *N* is finite, we can safely assume that the number of spike in the population is finite on finite time intervals $a.s$. (all spiking neuron model have a finite number of spikes on finite time interval $a.s$.). Between spikes, the evolution of the process is deterministic and easy to see. The more tricky increments are at spike times $\{t_{k}\}_{k \in \mathbb{Z}^{+}}$. At spike time $t_{k}$, we first draw a random sample $(\hat{u}(t_{k}), \hat{x}(t_{k}))$ according to Eq. (18f). Then we update *û* and *x̂*. Finally, we update *u*, *x*, *P*, *Q* and *R* according to Eqs. (18a)–(18g). Thus, $(u, x, P, Q, R, \hat{u}, \hat{x})$ is a jump process with random jump sizes, even when conditioned on $A^{N}$.
5. It is plausible that the second approximation step, in which the process $[y^{*},u^{*},x^{*},P^{*},Q^{*},R^{*},\hat{u}^{*},\hat{x}^{*}]$ is approximated by Eqs. (18a)–(18g), becomes valid for sufficiently large *N*. Here is a heuristic argument: $I_{\mathrm{syn}}^{N}(t)$ is a convolution of $y_{N}(t)$ with $\epsilon (t)$ (Eq. (6)). Assuming that *ϵ* is smooth and bounded, $I_{\mathrm{syn}}^{N}(t)$ is determined by the small time step integrals $\int _{t'}^{t'+\Delta t} y_{N}(t'')\,dt''$, for all $t'< t$ and $\Delta t > 0$ arbitrarily small. If the number of spikes in the interval $[t', t' + \Delta t]$ grows linearly with *N*, and if Δ*t* is small enough, under Assumption 1, we can see (heuristically) $\int _{t'}^{t'+\Delta t} y_{N}(t'')\,dt''$ as the sum of *αN* (*α* is some constant) $i.i.d$ random jumps scaled by $1/N$. Hence, by a central limit theorem type of argument, $\int _{t'}^{t'+\Delta t} y_{N}(t'')\,dt''$ only depends on the mean and the variance of the random jumps. In other words, even if the $[\hat{u}^{*}, \hat{x}^{*}]$ do not become Gaussian when *N* is large, Eqs. (18a)–(18g) should become accurate when *N* is sufficiently large because what matters (according to the above heuristic argument) is that $[\hat{u}, \hat{x}]$ has the right mean and variance. A rigorous proof of this heuristic argument would be of mathematical interest but goes beyond the scope of the present work.
6. By construction, the process $\{y(t)\}_{t>t_{0}}$ can be easily conditioned on the process $\{A^{N}(t)\}_{t>t_{0}}$: for any realization of the process $A^{N}(t)$, there is a well defined conditioned process $y_{|A^{N}}(t)$. This is a very practical feature because it means that we have a well defined approximation for any $A^{N}(t)$, stochastic or deterministic. Note that the process $A^{N}(t)$ does not need Assumption 1 to be satisfied to be well defined. Hence, $y_{|A^{N}}(t)$ is well defined even if Assumption 1 is not satisfied. Furthermore, this conditioning feature allows us to generalize the current mesoscopic approximation for feedforward networks to general mesoscopic networks as it will be explained in Sect. 3.

Instead of the Gaussian approximation, Eq. (18f), it is tempting to consider a first-order approximation, where $\hat{u}^{*}$ and $\hat{x}^{*}$ are approximated by the empirical means $u^{*}$ and $x^{*}$ neglecting their variance and covariance. Setting the covariance matrix in Eq. (18f) to zero yields the following.

#### First-order mean-field approximation

*We have*

19a $$\begin{aligned}& \frac{\mathrm{d}u}{\mathrm{d}t} =\frac{U_{0}-u}{\tau _{F}}+U\bigl(1-u\bigl(t^{-} \bigr)\bigr)A^{N}(t), \end{aligned}$$

19b $$\begin{aligned}& \frac{\mathrm{d}x}{\mathrm{d}t} =\frac{1-x}{\tau _{D}}- u\bigl(t^{-}\bigr) x \bigl(t^{-}\bigr) A^{N}(t), \end{aligned}$$

19c $$\begin{aligned}& y^{N}(t)\approx y(t) = u\bigl(t^{-}\bigr)x \bigl(t^{-}\bigr)A^{N}(t). \end{aligned}$$

This approximation is very similar to the classic mean-field equations derived for $N\rightarrow \infty $ by [30] except that it is driven by a sum of spike trains $A^{N}(t)=\frac{1}{N}\sum_{j=1}^{N} s_{j}(t)$ and not a continuous rate $r(t)$.

In this paper, we name Eqs. (18a)–(18g) the second-order mean-field theory (abbreviated second-order MF) and Eqs. (19a)–(19c) the first-order mean-field theory (abbreviated first-order MF).

In the rest of this work, we compare numerically the more sophisticated second-order MF to the simpler first-order MF. In this section, we focus on the case where the presynaptic spike trains $\{s_{j}\}_{j=1,\ldots ,N}$ are given by *N* independent Poisson processes with constant rate because in this case, Assumption 1 is satisfied. In Appendix B, we provide an efficient simulation algorithm for Eqs. (18a)–(18g).

**Figure 2:**
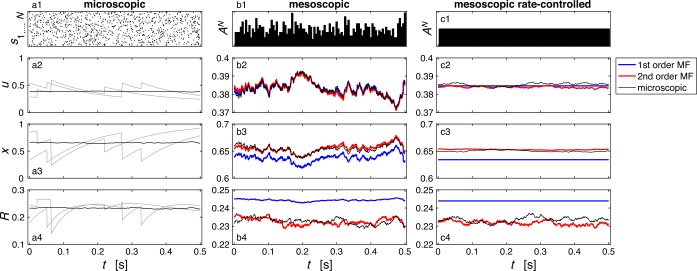
*Example of microscopic and mesoscopic synaptic dynamics for 200 presynaptic stationary Poisson neurons*. (**a1**) Raster plot of $N = 200$ presynaptic stationary Poisson neurons with rate 10 Hz. (**a2–4**) Trajectories of variables $u_{j}(t)$ and $x_{j}(t)$ and the resulting modulation factor $R_{j}(t) \equiv u_{j}(t)x_{j}(t)$ for two example neurons (gray lines). The black line shows the population averages $u(t)$, $x(t)$ and $R(t)$ calculated from Eqs. (15a)–(15c). (**b1**) Population activity $A^{N}(t)$ corresponding to the 200 spike trains shown in (**a1**). (**a2–4**) Trajectories of the mesoscopic variables $u(t)$, $x(t)$ and $R(t)$ predicted by the first- and second-order MF (blue and red, respectively) compared to the microscopic simulation (black) which correspond to the population averages shown on the left. Note that the y-axis scale is different in (**a2–4**) and (**b2–4**). In (**b4**), we see that, while finite-size fluctuations in *R* for the population average are reproduced by both first- and second-order MF, the first-order MF makes an error in predicting the mean. (**c1–4**) is the same as (**b1–4**) except that we force $A^{N}$ to be constant: while the $s_{j}$ are still Poisson spike trains with rate 10 Hz, they are generated such that $A^{N}$ is constant over time. This removes the effect of the finite-size fluctuations of $A^{N}$ on the finite-size fluctuations of the mesoscopic STP *u*, *x* and *R*. (**b2–4**) In contrast with the first-order MF, the second-order MF reproduces the residual finite-size fluctuations observed in the microscopic simulation. Synaptic parameters: $\tau _{\mathrm {D}}= 0.15\text{ s}$, $\tau _{\mathrm {F}}= 0.15\text{ s}$, $U = U_{0} = 0.2$. (**b1**) and (**c1**) are binned with bin size 0.005 ms

Trajectories of $u_{j}$, $x_{j}$ and $R_{j}$ as well as *u*, *x* and *R* obtained from a microscopic simulation are shown in Fig. 2. The mesoscopic variable *R* is tracked by the second-order MF dynamics with high accuracy (Fig. 2(b4)). The first-order MF also yields reasonable results, although a small deviation in the mean *R* over time is apparent (Fig. 2(b4)). This is consistent with previous findings [30], where it has been shown analytically that in the stationary case, relative correlations are small but significant. Note that the second-order MF distinguishes two sources of finite-size noise: noise that comes from the finite-size fluctuations of $A^{N}$ and second, noise that comes from the sampling of *û* and *x̂* at each spike. This second source of noise is absent in the first-order MF. In a numerical simulation with time step Δ*t*, it is possible to isolate this second source of noise: if $N \cdot \Delta t \cdot r$ (where *r* is the rate of the Poisson process) is a strictly positive integer *α*, we can choose, independently at each time step, *α* neurons uniformly across the *N* neurons and make them spike. This procedure generates *N* discretized Poisson spike trains of rate *r* with a “constant” population activity $A^{N}$ over time. Here, “constant” means that the time-discretized population activity $A^{N}(t)=n(t)/(N\Delta t)$ in the numerical simulation with time step Δ*t* is constant, i.e. the total number of spikes in $[t,t+\Delta t)$ is fixed. Note that in this case, the spike trains are not independent of each other but this does not affect our derivation. This procedure is followed in Fig. 2(c1–4): the first-order MF predicts noiseless STP dynamics whereas the second-order MF accurately reproduces the residual finite-size fluctuations.

**Figure 3:**
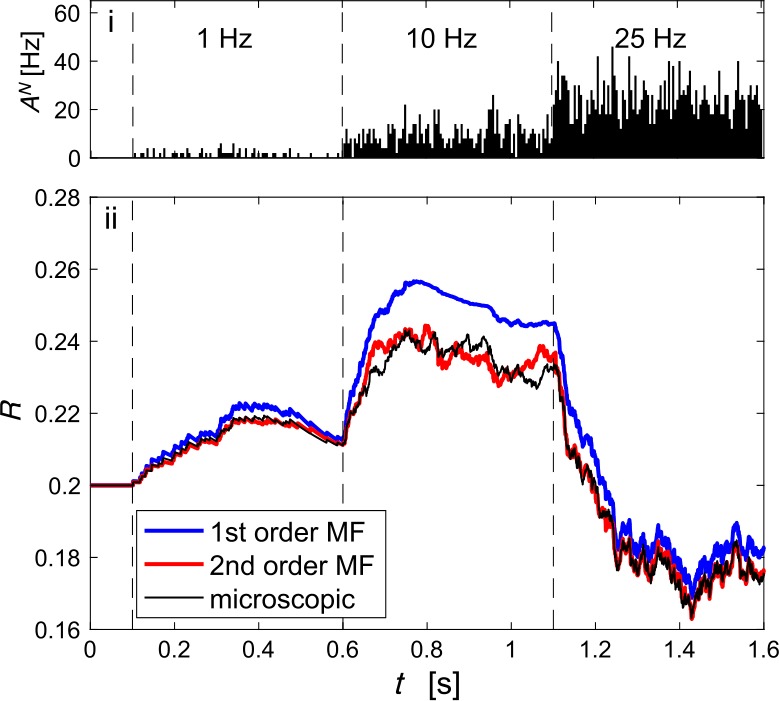
*Synaptic dynamics in response to step increments in the presynaptic firing rate*. (**i**) Population activity $A^{N}$ of 100 Poisson neurons when the presynaptic firing rate changes sharply from 0 Hz to 1, 10 and 25 Hz. (**ii**) Corresponding mesoscopic modulation factor *R* predicted by the first- and second-order MF (blue and red lines respectively) compared to the microscopic simulation (black line). Note that at 10 Hz the second-order MF corrects the overestimation in the mean of the first-order MF and reproduces finite-size fluctuations of amplitude similar to that of the population average. Synaptic parameters: $\tau _{D}=0.15\text{ s}$, $\tau _{F}=0.15\text{ s}$, $U=U_{0}=0.2$

The deviations of the first-order MF become more pronounced during non-stationary transients caused by stepwise increases of the rate of the Poisson process (Fig. 3). The response to step increases accurately traced by the second-order MF, but not by the first-order MF which neglects the correlations between $u_{j}$ and $x_{j}$.

### Statistics of the total postsynaptic input

**Figure 4:**
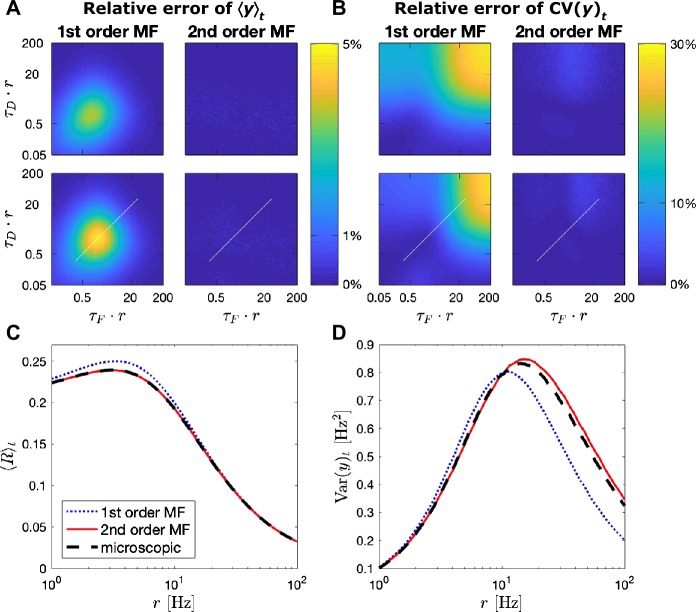
*First- and second-order statistics of the TPSI (y) for a presynaptic population of 100 stationary Poisson neurons*. (**A**), (**B**) Relative error of the mean TPSI over time $\langle y \rangle _{t}$ (**A**) and the coefficient of variation of the TPSI over time ($\text{CV}(y)_{t}$) (**B**) predicted by the first- and second-order MF (left and right column respectively) with respect to microscopic simulation, as a function of the synaptic parameters $\tau _{\mathrm {D}}$, $\tau _{\mathrm {F}}$ and for two values of *U* (with $U=U_{0}$); *U* is set to 0.5 on the upper row and 0.2 on the lower row. On the *x*- and *y*-axes, $\tau _{\mathrm {F}}\cdot r$ is a unitless quantity. In (**A**), the maximum relative error is 4.7% for the first-order MF and 0.3% for the second-order MF. In (**B**), the maximum relative error is 28.6% for the first-order MF and 4.0% for the second-order MF. As scaling $\tau _{\mathrm {F}}$ and $\tau _{\mathrm {D}}$ is equivalent to scaling the firing rate *r*, the relative error at different firing rates can be read moving along the diagonal (white line). (**C**) Mean modulating factor $\langle R \rangle _{t}$ over time predicted by the first- and second-order MF (dotted blue and solid red lines respectively) compared to microscopic simulations (dashed black line) as a function of the firing rate *r* for a specific set of synaptic parameters. (**D**) TPSI variance over time ($\operatorname{Var} (y )_{t}$) predicted by the first- and second-order MF compared to microscopic simulations as a function of the firing rate *r* for a specific set of synaptic parameters. Synaptic parameters used in (**C**)–(**D**) correspond to the white line in (**A**)–(**B**) and are: $\tau _{\mathrm {D}}= 0.3\text{ s}$, $\tau _{\mathrm {F}}= 0.3\text{ s}$ and $U =U_{0}=0.2$. Simulation time step is 0.5 ms

To compare microscopic and mesoscopic descriptions more systematically, we measured the first- and second-order statistics from simulations for varying parameters. At first, we computed the mean of the modulation factor *R* for the stationary process ($\langle s_{j}(t) \rangle = r = \text{const.}$) using the microscopic dynamics,

20 $$ \langle R\rangle =\bigl\langle u_{j}(t)x_{j}(t) \bigr\rangle , $$

where $\langle \cdot \rangle $ denotes the ensemble (trial) average, i.e. the average over realizations of the Poisson processes $s_{j}(t)$. The mean TPSI is proportional to the mean modulation factor because $\langle y\rangle =\frac{1}{N}\sum_{j}\langle u_{j}x_{j}\rangle \langle s_{j}\rangle =\langle R\rangle r$. This simple proportionality follows from the fact that $u_{j}(t)x_{j}(t)$ at time *t* is uncorrelated with $s_{j}(t)$ at the same time because of the Poisson statistic of the spike train and the update of variables after a spike; cf. Eqs. (7a) and (7b). As known from previous work [30], the mean modulation $\langle R \rangle $ of a facilitating synapse increases with increasing firing rate of presynaptic neurons when firing rates are small, and decreases again at high rates due to depression (Fig. 4(C)). The first-order MF shows small deviations in the mean modulation $\langle R\rangle $, which are removed by the second-order MF (Fig. 4(C)). A closer inspection of the full parameter regime reveals that the deviation of first-order MF never exceeds 5% (Fig. 4(A)). Therefore the stationary mean TPSI is sufficiently well explained by the first-order MF.

**Figure 5:**
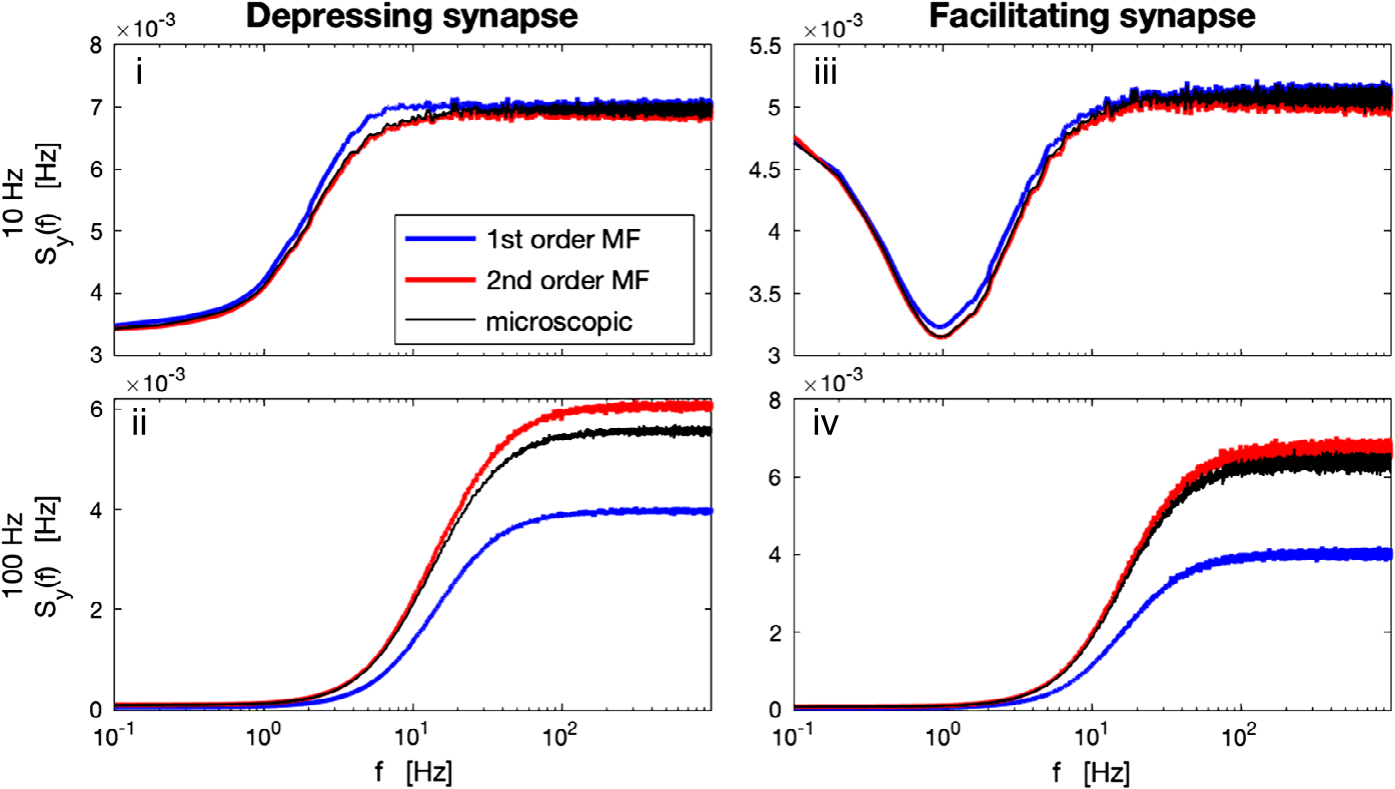
*Power spectral densities (PSD) of the TPSI given a presynaptic population of 200 stationary Poisson neurons*. PSD of the TPSI of a depressing synapse ((**i**) and (**ii**)) and a facilitating synapse ((**iii**) and (**iv**)), when the firing rate of the presynaptic neurons is 10 Hz ((**i**) and (**iii**)) and 100 Hz ((**ii**) and (**iv**)), predicted by the first- and second-order MF (blue and red lines respectively) compared microscopic simulations (black line). Each PSD is averaged over 5000 simulations and further smoothed using a moving average. Parameters for the depressing synapse: $\tau _{D}=0.1\text{ s}$, $\tau _{F}=0.05\text{ s}$, $U=U_{0}=0.5$. Parameters for the facilitating synapse: $\tau _{D}=0.1\text{ s}$, $\tau _{F}=0.7\text{ s}$, $U=U_{0}=0.1$. *A* in the upper panel is binned with bin size 0.005 ms

Also, we compared the statistics of fluctuations of the TPSI by measuring the respective power spectral densities (PSD). The PSD can be computed as

21 $$ S_{yy}(f)= \frac{\langle \overline{\tilde{y}(f)}\tilde{y}(f)\rangle }{T}, $$

where $\tilde{y}(f)=\int _{0}^{T}dt \exp (2\pi ift)y(t)$ denotes the Fourier transform of $y(t)$ for a finite but large enough time window *T*. We found that the second-order MF significantly better captured the variance (Fig. 4(D)) and the PSD (Fig. 5) of the stationary fluctuations than the first-order MF. A closer inspection of the coefficient of variation of the fluctuations, $\sqrt{\operatorname{Var} (y )}/\langle y\rangle $, over the full parameter space revealed that the first-order MF deviated up to 30% (especially for slow synaptic dynamics or high rates, Fig. 5(ii), (iv)), whereas the second-order model performed well in the whole parameter space (Fig. 4(B)). We should specify that the error of the first-order MF is negative, *i.e.* the first-order MF underestimates the coefficient of variation up to 30%. This comes from the fact that in the mesoscopic equations for the first-order MF equations (19a)–(19c), finite-size fluctuations of the mesoscopic variables *u* and *x* are ignored.

In conclusion, while mean responses for stationary cases are well captured by the first-order MF, the second-order MF gives a significantly better description of transient responses (Fig. 3) and fluctuations (Figs. 4(B), (D) and 5).

## Recurrent network with STP

### Microscopic model

As shown in our previous work [12], networks of multiple interacting homogeneous populations of spiking neurons, with static synapses, can be accurately predicted with a mesoscopic model. Incorporating the effect of STP in this general model using the mesoscopic approximations of Sect. 2.3 for the feedforward case is actually easy. This is due to the fact the mesoscopic approximation $y(t)$ (both the first-order MF and the second-order MF) can be conditioned on the population activity $A^{N}(t)$, as mentioned in Sect. 2.3. In order to illustrate this, we consider for simplicity the special case of a single population with recurrent connectivity. The network architecture is random with fixed in-degree $C=pN$, where *N* is the number of neurons in the population and *p* is the connection probability. The synaptic strength is constant with magnitude *w* (in mV). The TPSI $y_{i}(t)$ and the synaptic current $I_{\mathrm{syn},i}(t)$ driving the *postsynaptic* neuron *i* are related by Eq. (1) with $J=\frac{\tau _{\mathrm {m}}}{R_{\mathrm{m}}}Npw$. In this paper, we use a synaptic filtering kernel with instantaneous rise and exponential decay corresponding to the first-order kinetics

22 $$ \tau _{s} \frac{\mathrm{d}I_{\mathrm{syn},i}}{\mathrm{d}t} = -I_{ \mathrm{syn},i}+J y_{i}(t), $$

where $\tau _{s}$ is the synaptic filtering time constant. Importantly, the effect of STP is contained in the TPSI $y_{i}(t)=\frac{1}{C}\sum_{j\in \varGamma _{i}}u_{j}(t)x_{j}(t)s_{j}(t)$ via the synaptic variables $u_{j}$ and $x_{j}$ given by the dynamics Eq. (7a) and (7b). Here, $\varGamma _{i}$ denotes the index set of presynaptic neurons that connect to neuron *i*.

As our derivation of the mesoscopic theory of STP uses the assumption that neurons have Poisson statistics, we first apply our theory to Poisson rate neurons, which do not exhibit a dependence on spike history. Then, using the same setup, the theory will be applied to a network of generalized integrate-and-fire (GIF) neurons with pronounced spike-history effects.

#### Poisson rate model

The Poisson rate model is defined by a continuous variable $h_{i}(t)$, called input potential. The input potential of neuron *i* obeys the dynamics

23 $$ \tau _{\mathrm {m}}\frac{\mathrm{d}h_{i}}{\mathrm{d}t}=-h_{i}+\mu (t)+ R_{\mathrm{m}}I_{\mathrm{syn},i}(t), $$

where $\tau _{\mathrm {m}}$ represents the membrane time constant and $\mu (t)=V_{\mathrm{rest}}+R_{\mathrm{m}} I_{\mathrm{ext}}(t)$ is the total drive in the absence of synaptic input (constant resting potential $V_{\mathrm{rest}}$ and common external stimulus $I_{\mathrm{ext}}(t)$). In the fully-connected network, the synaptic current $I_{\mathrm{syn},i}(t)$ is the same for all neurons *i* and is given by Eq. (22).

In each time interval $[t,t+dt)$, spikes are drawn with probability $\lambda _{i}(t)\,dt$, where the firing rate $\lambda _{i}(t)$ depends on the input potential as follows:

24 $$ \lambda _{i}(t)=\varPhi \bigl(h_{i}(t) \bigr). $$

Here, we choose a sigmoidal transfer function of the form $\varPhi (h)=r_{\mathrm{max}}/ [1+\exp (-\beta (h-h_{0})) ]$ and $h_{0} = 0\mbox{ mV}$.

#### GIF model

The GIF model for the postsynaptic neuron dynamics is determined by the membrane potential $V_{i}(t)$, the dynamic threshold $\vartheta _{i}(t)$ and a conditional intensity $\lambda _{i}(t)$ for the stochastic spike generating mechanism. Here, the index $i=1,\ldots ,N$ represents the neuron label. Between the spikes, the membrane potential satisfies the dynamics

25 $$ \tau _{\mathrm {m}}\frac{\mathrm{d}V_{i}}{\mathrm{d}t}=-V_{i}+\mu (t)+R_{\mathrm{m}}I_{\mathrm{syn},i}(t), $$

where the quantities $\tau _{\mathrm {m}}$, $\mu (t)$, $R_{\mathrm{m}}$ and $I_{\mathrm{syn},i}(t)$ are the same as in the rate model above.

After a spike, the voltage is immediately reset to the potential $V_{\mathrm {r}}$, where it is clamped for an absolute refractory period $t_{\mathrm {ref}}=4\text{ ms}$.

Spikes are generated by a conditional intensity (hazard rate) of an exponential form:

26 $$ \lambda _{i}(t)= \textstyle\begin{cases} 0,& \text{if the last spike was emitted less than } t_{\mathrm {ref}}\text{ ago}, \\ c\exp (\frac{V_{i}(t)-V_{\mathrm {th}}}{\Delta _{u}} ),& \text{otherwise}, \end{cases} $$

where $V_{\mathrm {th}}$ is a constant. This means that the conditional intensity, and hence the probability $\lambda _{i}(t)\,dt$ to fire in the interval $[t,t+dt)$, depends on the momentary distance between the membrane potential and threshold. This completes the definition of the microscopic model.

### Mesoscopic mean-field model

As explained in [12], the random connectivity can be well approximated by a fully connected network ($C=N$), with rescaled synaptic weights *pw*, corresponding to a mean-field approximation. In the following, we shall therefore choose $p=1$ unless stated otherwise. In the mean-field approximation, the TPSI $y(t)$ and the synaptic current $I_{\mathrm{syn}}(t)$ do not depend on the identities *j* of the postsynaptic neurons and are related by Eq. (1) with $J=\frac{\tau _{\mathrm {m}}}{R_{\mathrm{m}}}Npw$. For the case of exponential synapses, the synaptic current reads

27 $$ \tau _{s} \frac{\mathrm{d}I_{\mathrm{syn}}}{\mathrm{d}t} = -I_{ \mathrm{syn}}+J \hat{u}\bigl(t^{-}\bigr)\hat{x} \bigl(t^{-}\bigr)A^{N}(t), $$

where *û* and *x̂* obey the mean-field equations (18a)–(18g).

As shown in [12], the population activity $A^{N}(t)$ can be determined by a single mesoscopic variable, the instantaneous rate $r(t)$, whose dynamics depends on the microscopic model and the history of the population activity $\mathcal{H}_{t}=\{A(t')|t'< t\}$. Specifically, $A^{N}(t)$ is given by the normalized spike train

28 $$ A^{N}(t)=\frac{1}{N}\frac{dn^{N}(t)}{dt}= \frac{1}{N}\sum_{k\in \mathbb{Z}^{+}}\delta (t-{t_{k}}), $$

where $n^{N}(t)$ is a counting process with (conditional) intensity $\hat{\lambda }(t)=Nr(t)$ representing the total number of spikes in the population up to time *t*. The second equality means that $A^{N}(t)$ is proportional to a spike train with spike times $t_{k}$ generated with rate $Nr(t)$. This is similar to the superposition of Poisson spike train in the feedforward case, Sect. 2, where the pooled spike train also exhibits the rate $Nr(t)$.

#### Poisson rate model

In the Poisson rate model, the rate $r(t)$ is given by

29 $$\begin{aligned}& r(t) =\varPhi \bigl(h(t) \bigr), \end{aligned}$$

30 $$\begin{aligned}& \tau _{\mathrm {m}}\frac{\mathrm{d}h}{\mathrm{d}t} =-h+\mu (t)+R_{\mathrm{m}} I_{ \mathrm{syn}}(t). \end{aligned}$$

Importantly, the coupling to the STP dynamics is contained in the synaptic current $I_{\mathrm{syn}}$ governed by Eq. (27) and the synaptic mean-field dynamics given by Eqs. (18a)–(18g).

#### GIF population model

For the model with spike-history dependence, the rate $r(t)$ is obtained from an integral over refractory states. A possible representation of the neuronal refractory state is given by the time *τ* since the last spike (“age of the neurons”; an alternative representation in terms of the last spike times $\hat {t}=t-\tau $ is given in Appendix D). In the following, we assume that the process is initialized at $t_{0}=-\infty $. Given the distribution of ages in the population, $q(\tau ,t)$, the rate at time *t* results from [12],

31 $$ r(t)= \int _{0}^{\infty }\lambda (t,\tau )q(\tau ,t) \,\mathrm{d}\tau +\varLambda (t) \biggl( 1- \int _{0}^{\infty }q(\tau ,t) \,\mathrm{d}\tau \biggr), $$

where the density $q(\tau ,t)$ evolves according to the first-order partial differential equation with time-dependent boundary condition at $\tau =0$:

32 $$ (\partial _{t}+\partial _{\tau })q =- \lambda (t,\tau )q ,\qquad q (0,t)=A (t). $$

Here, $A^{N}(t)$ is given by Eq. (28). In Eqs. (31) and (32), the functions *λ* and *Λ* are given by

33 $$ \lambda (t,\tau )=c\exp \biggl(\frac{V(t,\tau )-{V_{\mathrm {th}}}}{\Delta _{u}} \biggr),\qquad \varLambda (t)= \frac{\int _{0}^{\infty }\lambda (t,\tau )W(t,\tau ) \, \mathrm{d}\tau }{\int _{0}^{\infty }W(t,\tau )\, \mathrm{d}\tau }, $$

where *V* and *W* are dynamical variables that obey the following dynamics: The age-dependent membrane potential $V(\tau ,t)$ and variance function $W(\tau ,t)$ follow the first-order partial differential equations

34 $$\begin{aligned}& (\partial _{t}+\partial _{\tau })V =- \frac{V -\mu }{\tau _{\mathrm {m}}}+ \frac{R_{\mathrm{m}}}{\tau _{\mathrm {m}}} I_{\mathrm{syn}}(t), \end{aligned}$$

35 $$\begin{aligned}& (\partial _{t}+\partial _{\tau })W =- \lambda (t,\tau )[2W -q ], \end{aligned}$$

with boundary conditions $V(t,0)=V_{\mathrm {r}}$ and $W(t,0)=0$. The coupling to the STP mean-field dynamics, Eqs. (18a)–(18g), is contained in the synaptic current $I_{\mathrm{syn}}$ governed by Eq. (27), which influences the voltage $V(t, \tau )$ (Eq. (34)), and hence changes $\lambda (t, \tau )$ and $\varLambda (t)$.

The population equations (31)–(35) have been efficiently integrated numerically by the algorithm presented in [12]. The numerical integration of the STP mean-field dynamics is given in Appendix B.

### Recurrent network of Poisson rate neurons—microscopic vs. mesoscopic simulations

#### Finite-size noise induced population spikes

An interesting example of collective neural dynamics, potentially linked to synaptic depression, is the phenomenon of population spikes in cultured neural networks [37]. We asked whether population spikes, a brief period of high average population activity, can be explained by our finite-size population theory with STP. As in previous work [37], we considered a single excitatory population endowed with STP. The mesoscopic mean-field equations allowed us to choose parameters of this model such that the macroscopic mean-field dynamics ($N \rightarrow \infty $, see Eqs. (56a)–(56e)) is in an excitable regime for the second-order MF but not for the first-order MF. Here excitable regime means that the macroscopic dynamics converges to an equilibrium point if the total drive remains below a certain threshold. However, if the threshold is exceeded (e.g. by a brief excitable stimulus or an increase of recurrent synaptic excitation), the activity rises rapidly to large values due to the positive feedback of recurrent excitation. The explosive rise of the activity is terminated by the beginning of synaptic depression, which acts as negative feedback and ultimately wins over recurrent excitation. As a result of the initial excitation, the population activity may show population spikes similar to action potential in other excitable systems such as single neurons.

**Figure 6:**
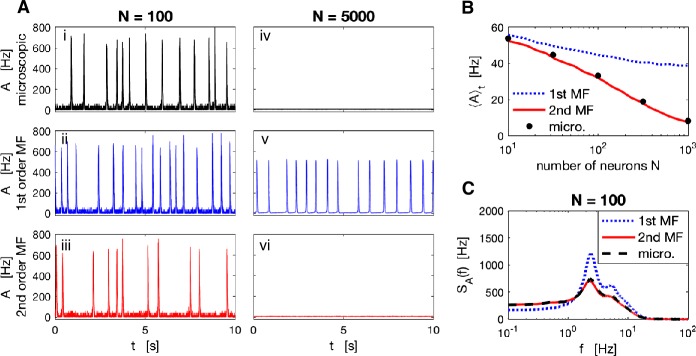
*Recurrent network of Poisson neurons with finite-size noise generating irregular population spikes*. (**A**) For small population size, $N=100$, microscopic (**i**) as well as first- (**ii**) and second-order (**iii**) MF dynamics exhibit irregular population spikes. For large population size, $N=5000$, and hence weak finite-size noise, population spikes cease in microscopic (**iv**) and second-order MF dynamics (**vi**) consistent with an excitable dynamics, whereas the first-order MF approximation (**v**) wrongly predicts regular population spikes corresponding to an underlying oscillatory (limit-cycle) dynamics. (**B**) Time-averaged population activity decreases with increasing population size indicating a decrease of population spike frequency. The prediction of the second-order MF is accurate across all population sizes, which is not the case with the first-order MF, especially for large populations. (**C**) PSD of population activities for $N=100$ neurons. Model parameters are detailed in Appendix E. Superscript (^*N*^) of $A^{N}$ is omitted in the legends

As expected for an excitable system driven by noise [42], the population activity exhibits irregular population spikes if the population size is small (here $N=100$), i.e. if finite-size noise is sufficiently strong (Fig. 6(A)(i)–(iii)). In our case, the drive that causes population spikes originates from finite-size fluctuations as expressed by the stochastic terms in Eq. (28). For $N = 100$, the second-order MF accurately predicts the mean activity (Fig. 6(B)) and power spectrum (Fig. 6(C)) of the full microscopic simulation whereas the first-order MF deviates quantitatively. Importantly, in the limit of large population size, population spikes vanish in the second-order MF theory consistent with microscopic simulations (Fig. 6(A)(vi), (iv), $N=5000$). In marked contrast to microscopic simulations, highly regular population spikes persist even for $N=5000$ in the first-order MF approximation corresponding to a deterministic limit-cycle dynamics (Fig. 6(A)(v)).

In summary, the second-order MF approximation accurately reproduces the qualitative behavior and the mean and the power spectrum of excitatory networks of Poisson neurons with synaptic STP. The statistical properties of the first-order MF dynamics exhibit quantitative deviations of statistical properties and in some cases fails to reproduce the qualitative behavior if the system is poised near a bifurcation. The large discrepancies between the first-order MF and the microscopic model in the example we show (Fig. 6) are mainly caused by the error in the mean modulation factor *R*. Indeed, for our choice of $\tau _{D} = \tau _{F} = 1\text{ s}$, correlations between $u_{j}$ and $x_{j}$ are relatively strong but are neglected by the first-order MF. We note that this error appears already for the deterministic (i.e. $N\rightarrow \infty $) dynamics. Inaccuracies in the correct description of finite-size noise in the first-order MF model may yield additional sources of errors.

**Figure 7:**
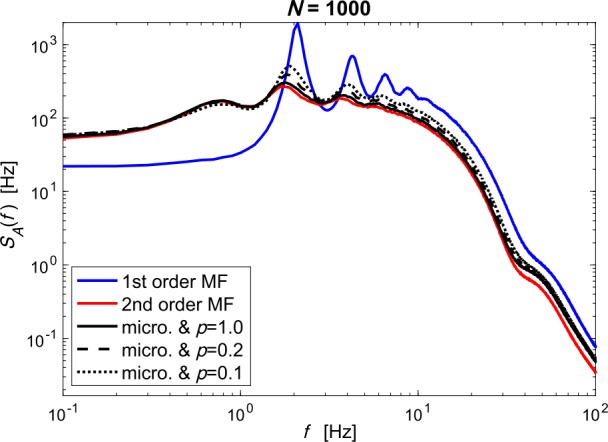
*PSD of population activities with different connection probabilityp(same setup as Fig. **6**)*. A microscopic network of $N=1000$ neurons is simulated with three different connection probabilities: $p=1.0, 0.2 \text{ and } 0.1$. Synaptic weights *w* are set such that the rescaled synaptic weights *pw* is constant. These three microscopic networks share the same mean-field approximation. Notice that the second-order MF is more accurate that the first-order MF even if the connection probability is reduced. Here, the model parameters are slightly modified from Fig. 6 to ensure that population spikes occur in the microscopic simulation 1000 neurons. Parameters are detailed in Appendix E. Superscript (^*N*^) of $A^{N}$ is omitted in the legends

Furthermore, the second-order MF remains accurate when the connection probability *p* of the network is smaller than 1 (given that the number *pN* of incoming synapses per neuron is roughly greater than 100). When *p* is smaller than 1, we argue in Sect. 3.2 that the microscopic network can be approximated by a fully-connected network with rescaled synaptic weights (mean-field approximation). We test this claim numerically in Fig. 7: using the same setup as in Fig. 6, for a population size of $N=1000$ neurons, we show that the second-order MF yields more accurate prediction than the first-order MF even when the connection probability is reduced down to 0.1.

#### Bistable switching between Up and Down states induced by finite-size fluctuations

**Figure 8:**
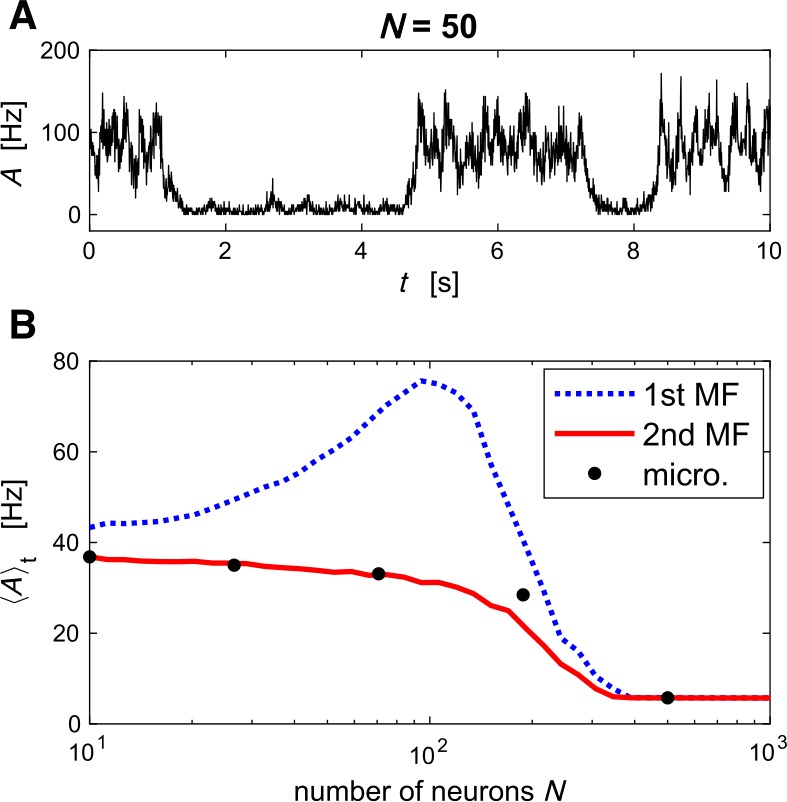
*Switchings between Up and Down states induced by finite-size fluctuations in a recurrent networks of Poisson neurons with STP*. (**A**) Microscopic simulation of a fully connected network of $N=50$ neurons. The population activity shows switches between Up and Down states. (**B**) Time-averaged population activity $\langle A^{N} \rangle _{t}$ (which depends on the probabilities of being in the Up and Down states) for different population sizes. Microscopic simulations (black dots) are compared to mesoscopic simulations with the first- and second-order MF equations (blue and red lines, respectively). Note that, for $N\sim 100$ neurons, the first-order MF approximation predicts significantly larger $\langle A^{N} \rangle _{t}$ indicating larger residence times in the Up state due to underestimation of finite-size noise. The same set of parameters are used in (**A**) and (**B**) and details are in Appendix E. Superscript (^*N*^) of $A^{N}$ is omitted in the legends

Another collective phenomenon in neural networks is multistability. In the presence of finite-size noise, systems with multistable behavior exhibit switches between different attractor states [12, 43, 44]. In particular, bistable neural systems driven by noise support stochastic switches between high and low population activity (“Up and Down states”) [12, 31, 45, 46]. As a starting point of our simulations of Up and Down states, following [31], we use an excitatory population with synaptic depression in the bistable regime. The qualitative behavior of the microscopic model exhibiting Up and Down states is captured by both first- and second-order MF (Fig. 8). A closer look at the mean firing rate, which is mainly determined by the ratio of the time spent in the Up or Down state, reveals that the first-order MF dynamics predicts significantly longer residence times in the Up state (Fig. 8(B)). In contrast, the second-order MF approximation accurately matches the simulation of the microscopic model. In this example we have chosen $\tau _{D} \gg \tau _{F}$ such that the correlations between $u_{j}$ and $x_{j}$ are negligible. As the consequence, the mean modulation factors *R* predicted by the first- and second-order MF theories, and hence the mean TPSI $\langle y\rangle $, are almost equal (cf. Fig. 4(A)). The error made by the first-order MF approximation mainly results from an incorrect description of finite-size fluctuations: at high firing rate (Up state), finite-size fluctuations are largely underestimated in the first-order MF dynamics as mentioned in Sect. 2.4. The weaker noise implies longer residence times in the Up state. This example highlights the relevance of the fluctuation statistics provided by the second-order MF approximation.

### Recurrent network of GIF neurons—microscopic vs. mesoscopic simulations

**Figure 9:**
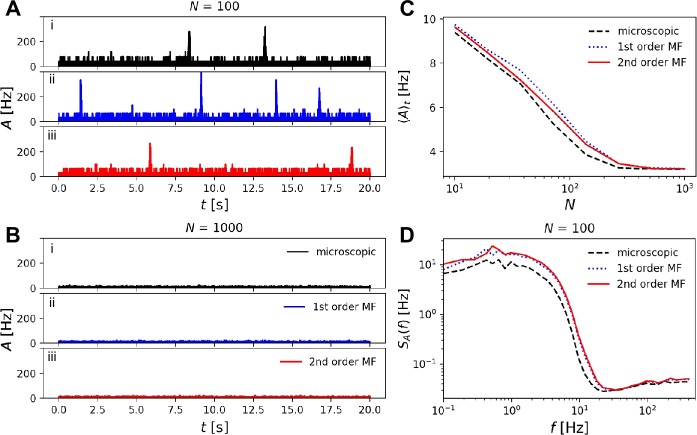
*Finite-size noise induced population spikes in recurrent networks of GIF neurons*. (**A**) Population activity of a fully connected network of $N=100$ neurons exhibiting irregular population spikes (microscopic simulation (**A**)(**i**), first- and second-order MF theory (**A**)(**ii**) and (**A**)(**iii**), respectively). (**B**) Same as (**A**) but with $N=1000$. Finite-size fluctuations are not strong enough to elicit population spikes. (**C**) Time-averaged population activity $\langle A^{N} \rangle _{t}$ (which depends on the frequency of population spikes) for different *N*. (**D**) Power spectral densities of the population activity for a network of 100 neurons. The same set of parameters is used in all panels and is detailed in Appendix E. Superscript (^*N*^) of $A^{N}$ is omitted in the legends

As a final demonstration of the mesoscopic MF theory with STP, we consider an excitable regime generating population spikes as in Sect. 3.3.1 but with more realistic neurons described by a GIF spiking neuron model (Sects. 3.1.2 and 3.2.2). Because of spike-history dependencies such as refractoriness, spike arrivals at synapses are no longer Poisson processes. Hence, the Poisson assumption of first- and second-order MF theories is not fulfilled anymore for recurrent GIF networks. Nevertheless, it is interesting to see whether population spikes can still be captured by the mesoscopic MF equations. To this end, we simulated the recently developed mesoscopic population equations for populations of GIF neurons [12] given by Eqs. (22), (31)–(35) extended by the MF equations for STP, Eqs. (18a)–(18g). The full MF theory qualitatively reproduces population spikes at small population sizes (Fig. 9(A)) and their extinction for large populations (Fig. 9(B)). Both the mean (Fig. 9(C)) and fluctuation statistics (Fig. 9(D)) are roughly captured by the MF equations albeit with small deviations from the microscopic simulation. However, a clear advantage of 2nd vs. first-order approximation is not apparent. This indicates that the second-order approximation does not necessarily yield a better approximation for networks of non-Poisson neurons and that the computationally simpler first-order MF model might be preferable in spiking neural networks with strong spike-history effects.

## Discussion

We have derived stochastic mean-field (MF) equations that capture the effect of synaptic short-term plasticity (STP) at the level of populations. These equations generalize previous MF theories for deterministic population rates [4, 30, 31] to the case of finite-size populations with stochastic population rates (mesoscopic description). The mesoscopic STP dynamics is compatible with a recent mesoscopic population model [12], which has been originally derived for static synapses. The mesoscopic MF dynamics of STP can thus be easily included into existing mesoscopic models. We find that a first-order mean-field approximation that accounts for stochastic rates but neglects correlations between facilitation and depression variables (as in [47]) approximates well the mean stationary input. This mean input is slightly improved by a second-order approximation, which accounts for correlations but neglects third and higher-order cumulants. The main strength of the second-order MF theory lies in the prediction of fluctuations and transient responses of the STP variables. We have shown that population spikes and UP and Down state switches in a one-population model with synaptic depression can be well described by the extended mesoscopic model. In particular, the second-order MF equations accurately replicate simulations of a network of Poisson neurons coupled via dynamic synapses. For networks of GIF spiking neurons the agreement is less accurate but still captures the qualitative collective dynamics.

In simulations of neuronal populations with STP, our mesoscopic mean-field model yields a considerable reduction of computational complexity. Compared to a network with static synapses, each neuron is endowed with two additional variables $u_{j}$ and $x_{j}$ that capture the effect of dynamic synapses onto its postsynaptic target neurons. In a single population of *N* neurons and connection probability *p*, a microscopic simulation thus requires the numerical integration of $2pN$ additional equations. By contrast, a simulation of the mesoscopic model only needs 4 additional equations per population. Thus we expect that our extended mesoscopic dynamics offers a significant speed up of large-scale simulations of cortical circuits with dynamic synapses [7].

An interesting question that has been studied theoretically [27–29] is how STP affects information transmission through a large ensemble of dynamic synapses. Our reduction of a synaptic ensemble to a four-dimensional nonlinear mean-field dynamics offers a mathematical framework to derive approximate analytical expressions for measures of information transmission. Analysing information processing capabilities of STP in the context of our mean-field theory is an interesting topic for future studies.

We have employed the deterministic STP model of Tsodyks and Markram [30]. While the resulting mean-field equations hold for this specific model, the same approach can be applied straightforwardly to other deterministic models of STP (e.g. [21, 48]). It is less obvious how to treat *stochastic* models of STP. Biological synapses are highly stochastic owing to the small number of synaptic vesicles that are randomly released upon spike arrival. This includes a finite probability of transmission failure. Using stochastic models of STP, it has been shown that synaptic stochasticity has a strong impact on information transmission [28] and postsynaptic neural responses [49]. On the population level, it seems to be feasible to treat this source of randomness in a similar manner as we did in Sect. 2.3. A generalization to a mesoscopic STP model that is applicable for stochastic synapses, will be an important subject for further studies.

The mean-field equations for the STP dynamics have been derived under the assumption that presynaptic spike trains are, loosely speaking, Poisson (Assumption 1). We have tested the mean-field equations in a feedforward setup and a recurrent network of Poisson rate units, where Assumption 1 holds true, and we found excellent agreements with microscopic simulations. For the application to recurrent networks of generalized integrate-and-fire (GIF) neurons in Sect. 3.4, Assumption 1 is not fulfilled because of refractoriness and other spike-history dependencies of single neurons [50, 51]. Despite the non-Poisson (colored-noise) statistics of spike trains in integrate-and-fire networks, a Poisson (white-noise) assumption is commonly used in mean-field theories as a “first-order approximation” [52]. In a similar spirit, we here simply assumed that synaptic input can be treated as a Poisson process so as to apply our MF theory for STP to networks of GIF neurons. For a simple one-population model with excitatory synaptic connections and STP that exhibits non-trivial dynamics in the form of population spikes, we have shown that the MF equations reproduce the qualitative behavior. This indicates that the MF theory may be valid beyond networks of Poisson neurons. A theoretical analysis of the effect of non-Poisson inputs and the region of validity of the present MF model is beyond the scope of the present paper and remains to be studied. To this end, theoretical approaches to treat dynamic synapses driven by renewal processes [53–55] might be a promising starting point.

Note that although the derivation of the mesoscopic mean-field approximation is systematic we do not obtain any mathematical guarantee that the process $y(t)$ is close (in any sense) to the process $y^{N}(t)$ we want to approximate. In the case where the spike trains $\{s_{j}\}_{j=1, \ldots , N}$ are *N* independent Poisson processes of rate $\lambda (t)$, it might be possible to prove that the processes $y(t)$ and $y^{N}(t)$ converge to the same diffusion approximation as *N* tends to infinity. Obtaining such a proof would be, however, challenging because in our case, we do not know around which mean value the process fluctuates, i.e. we do not know the deterministic $N \to \infty $ limit. This is due to the product $u_{j} x_{j}$ in Eq. (7b) and the fact that we do not have a closed form expression for the limit of $\frac{1}{N}\sum_{j=1}^{N} u_{j} x_{j}$ when $N \to \infty $. Note that if one considers purely facilitating (or purely depressing) synapses, the deterministic $N \to \infty $ limit can be computed (see [32]). Even in the case of purely facilitating or depressing synapses, deriving the diffusion approximation for the evolution of the mesoscopic variables $u(t)$ or $x(t)$ would be non-trivial because the jump sizes are modulated by the microscopic $u_{j}$ and $x_{j}$ (see Eqs. (38a)–(38b)). These are two independent reasons why standard techniques from the fluid limit literature (see [38–40]) cannot be applied directly. Also, one clear advantage of our current approach over the diffusion approximation approach is that our mesoscopic approximation can be conditioned on the process $A^{N}(t)$, i.e. it can be conditioned on any sequence of spike times $\{t_{k}\}_{k \in \mathbb{Z}^{+}}$.

In our previous work [12], we have developed a mean-field theory of neuronal populations that incorporates spike-history dependencies, such as refractoriness and adaptation, and finite-size fluctuations in a consistent manner. By adding another important feature—synaptic short-term plasticity—we have here made a further step towards a microscopically-grounded mesoscopic population model of a cortical circuit.

## Appendix 1: Proof of Lemma 2.1

To derive the system of equations (16a)–(16c), it is useful to rewrite the microscopic synaptic dynamics Eqs. (11a)–(11b) in differential form:

36 $$\begin{aligned}& du_{j} =\frac{U_{0}-u_{j}}{\tau _{F}}\,dt+U \bigl(1-u^{-}_{j}\bigr)\,dn_{j}(t), \end{aligned}$$

37 $$\begin{aligned}& dx_{j} =\frac{1-x_{j}}{\tau _{D}}\,dt-u^{-}_{j}x^{-}_{j}\,dn_{j}(t). \end{aligned}$$

Here, $dn_{j}(t)$ denotes the increment of the counting process $n_{j}(t)=\int ^{t}_{0}s_{j}(t') \,dt'$ at the *j*th synapse and $u^{-}_{j}$ is a shorthand for the left limit. The stochastic differential equations for the mesoscopic quantities $u^{N}(t)$ and $x^{N}(t)$ are then

38a $$\begin{aligned} du^{N}&=\frac{U_{0}-u^{N}}{\tau _{F}}\,dt+U \frac{1}{N}\sum^{N}_{j=1} \bigl(1 - u_{j}^{-}\bigr)\,dn_{j}(t), \end{aligned}$$

38b $$\begin{aligned} dx^{N}&=\frac{1-x^{N}}{\tau _{D}}\,dt- \frac{1}{N}\sum^{N}_{j=1}u_{j}^{-}x_{j}^{-}\,dn_{j}(t). \end{aligned}$$

With some calculations, we can write similar equations for $P^{N}(t)$, $Q^{N}(t)$ and $R^{N}(t)$. For instance, to compute $dP^{N}$ we need to evaluate $d(u_{j}^{2})$. Using Itô formula for jump processes, we get

$$ d \bigl(u_{j}(t)^{2} \bigr)=2u_{j}(t) \frac{U_{0}-u_{j}(t)}{\tau _{F}}\,dt+ \bigl(u_{j}(t)^{2} - u_{j}\bigl(t^{-}\bigr)^{2} \bigr)\,dn_{j}(t). $$

Similarly, we write

$$\begin{aligned}& d \bigl(x_{j}(t)^{2} \bigr)=2x_{j}(t) \frac{1-x_{j}(t)}{\tau _{D}}\,dt+ \bigl(x_{j}(t)^{2} - x_{j}\bigl(t^{-}\bigr)^{2} \bigr)\,dn_{j}(t), \\& \begin{aligned} d \bigl(u_{j}(t)x_{j}(t) \bigr)&=u_{j}(t) \frac{1-x_{j}(t)}{\tau _{D}}\,dt+x_{j}(t) \frac{U_{0}-u_{j}(t)}{\tau _{F}}\,dt \\ &\quad {} + \bigl(u_{j}(t)x_{j}(t) - u_{j} \bigl(t^{-}\bigr)x_{j}\bigl(t^{-}\bigr) \bigr)\,dn_{j}(t). \end{aligned} \end{aligned}$$

We now compute the jump terms:

39 $$\begin{aligned}& \bigl(u_{j}(t)^{2} - u_{j} \bigl(t^{-}\bigr)^{2} \bigr)\,dn_{j}(t) = U \bigl(1-u_{j}\bigl(t^{-}\bigr)\bigr)\bigl[(2-U)u_{j} \bigl(t^{-}\bigr)+U\bigr] \,dn_{j}(t), \end{aligned}$$

40 $$\begin{aligned}& \bigl(x_{j}(t)^{2} - x_{j} \bigl(t^{-}\bigr)^{2} \bigr)\,dn_{j}(t) =u_{j} \bigl(t^{-}\bigr)x_{j}\bigl(t^{-} \bigr)^{2}\bigl(2-u_{j}\bigl(t^{-}\bigr) \bigr)\,dn_{j}(t), \end{aligned}$$

41 $$\begin{aligned}& \bigl(u_{j}(t)x_{j}(t) - u_{j} \bigl(t^{-}\bigr)x_{j}\bigl(t^{-}\bigr) \bigr)\,dn_{j}(t) =x_{j}\bigl(t^{-}\bigr) \bigl[U\bigl(1-u_{j}\bigl(t^{-}\bigr)\bigr)^{2}-u_{j} \bigl(t^{-}\bigr)^{2} \bigr]\,dn_{j}(t). \end{aligned}$$

Summing the differentials $d(u_{j}^{2})$, $d(x_{j}^{2})$ and $d(u_{j}x_{j})$ over all *N* synapses, we obtain

42a $$\begin{aligned}& dP^{N} =2\frac{U_{0}u^{N}-P^{N}}{\tau _{F}}\,dt+\sum_{j=1}^{N} U\bigl(1-u_{j}^{-}\bigr)\bigl[(2-U)u_{j}^{-}+U \bigr] \frac{dn_{j}(t)}{N}, \end{aligned}$$

42b $$\begin{aligned}& dQ^{N} =2\frac{x^{N}-Q^{N}}{\tau _{D}}\,dt- \sum_{j=1}^{N} u_{j}^{-}\bigl(x_{j}^{-} \bigr)^{2}\bigl(2-u_{j}^{-}\bigr) \frac{dn_{j}(t)}{N}, \end{aligned}$$

42c $$\begin{aligned}& dR^{N} = \biggl( \frac{U_{0}x^{N}-R^{N}}{\tau _{F}}+ \frac{u^{N}-R^{N}}{\tau _{D}} \biggr)\,dt+\sum_{j=1}^{N} x_{j}^{-} \bigl[U\bigl(1-u_{j}^{-} \bigr)^{2}-\bigl(u_{j}^{-} \bigr)^{2} \bigr]\frac{dn_{j}(t)}{N}. \end{aligned}$$

For notational simplicity, we have derived Eqs. (38a)–(38b) and (42a)–(42c) for the variables $[u^{N},x^{N},P^{N},Q^{N},R^{N}]$ defined by Eqs. (15a)–(15c) starting from microscopic variables $u_{j}$ and $x_{j}$ defined by Eqs. (7a)–(7b). A completely analogous derivation can be performed for the variables $[u^{*},x^{*},P^{*},Q^{*},R^{*}]$ defined by Eqs. (16a)–(16c) starting from the corresponding microscopic variables $u_{j}^{*}$ and $x_{j}^{*}$ defined by Eqs. (11a)–(11b). For the variables marked with asterisk, we thus have

43a $$\begin{aligned}& du^{*} =\frac{U_{0}-u^{*}}{\tau _{F}}\,dt+U \frac{1}{N}\sum^{N}_{j=1} \bigl(1 - u_{j}^{*}\bigr)\,dn_{j}^{*}(t), \end{aligned}$$

43b $$\begin{aligned}& dx^{*} =\frac{1-x^{*}}{\tau _{D}}\,dt- \frac{1}{N}\sum^{N}_{j=1}u_{j}^{*}x_{j}^{*}\,dn_{j}^{*}(t), \end{aligned}$$

43c $$\begin{aligned}& dP^{*} =2\frac{U_{0}u^{*}-P^{*}}{\tau _{F}}\,dt+\sum_{j=1}^{N} U\bigl(1-u_{j}^{*}\bigr)\bigl[(2-U)u_{j}^{*}+U \bigr] \frac{dn_{j}^{*}(t)}{N}, \end{aligned}$$

43d $$\begin{aligned}& dQ^{*} =2\frac{x^{*}-Q^{*}}{\tau _{D}}\,dt- \sum_{j=1}^{N} u_{j}^{*}x_{j}^{*2} \bigl(2-u_{j}^{*}\bigr) \frac{dn_{j}^{*}(t)}{N}, \end{aligned}$$

43e $$\begin{aligned}& dR^{*} = \biggl( \frac{U_{0}x^{*}-R^{*}}{\tau _{F}}+ \frac{u^{*}-R^{*}}{\tau _{D}} \biggr)\,dt+\sum_{j=1}^{N} x_{j}^{*} \bigl[U\bigl(1-u_{j}^{*} \bigr)^{2}-u_{j}^{*2} \bigr] \frac{dn_{j}^{*}(t)}{N}, \end{aligned}$$

where $u_{j}^{*}$ and $x_{j}^{*}$ are shorthands for $u_{j}^{*}(t^{-})$ and $x_{j}^{*}(t^{-})$, respectively.

The sums over the weighted spike counts $dn_{j}^{*}$ cannot be expressed as a deterministic function of the mesoscopic spike count $dn^{N}=\sum_{j}n_{j}^{*}(t)$. However, we can make use of the fact that in an infinitesimally small time interval *dt* at most one spike can occur and contribute to the sums, which thereby simplify considerably: $dn_{j}^{*}$ is either zero for all *j* (i.e. $dn^{N}=0$), or there exists one and only one *j* for which $dn_{j}^{*}=1$ (in this case $dn^{N}=1$). This implies that we can write for any function $g(u_{j}^{*},x_{j}^{*})$ multiplying $dn_{j}^{*}$

44 $$ \frac{1}{N}\sum_{j}g \bigl(u_{j}^{*},x_{j}^{*} \bigr)\,dn_{j}^{*}=g\bigl(u_{j^{*}(k)}^{*},x_{j^{*}(k)}^{*} \bigr) \frac{dn^{N}}{N}=g\bigl(\hat{u}^{*},\hat{x}^{*} \bigr)\frac{dn^{N}}{N}, $$

where $j^{*}(k)$ is the random synapse index at time $t_{k}$ and $(\hat{u}^{*}(t), \hat{x}^{*}(t))$ is given by Eq. (12). Applying relation (44) to Eqs. (43a)–(43e), we obtain

45a $$\begin{aligned}& du^{*} =\frac{U_{0}-u^{*}}{\tau _{F}}\,dt+U \bigl(1-\hat{u}^{*} \bigr)\frac{dn^{N}(t)}{N}, \end{aligned}$$

45b $$\begin{aligned}& dx^{*} =\frac{1-x^{*}}{\tau _{D}}\,dt-\hat{u}^{*} \hat{x}^{*} \frac{dn^{N}(t)}{N}, \end{aligned}$$

45c $$\begin{aligned}& dP^{*} =2\frac{U_{0}u^{*}-P^{*}}{\tau _{F}}\,dt+U\bigl(1- \hat{u}^{*}\bigr)\bigl[(2-U) \hat{u}^{*}+U\bigr] \frac{dn^{N}(t)}{N}, \end{aligned}$$

45d $$\begin{aligned}& dQ^{*} =2\frac{x^{*}-Q^{*}}{\tau _{D}}\,dt-\hat{u}^{*} \hat{x}^{*2}\bigl(2- \hat{u}^{*}\bigr) \frac{dn^{N}(t)}{N}, \end{aligned}$$

45e $$\begin{aligned}& dR^{*} = \biggl( \frac{U_{0}x^{*}-R^{*}}{\tau _{F}}+ \frac{u^{*}-R^{*}}{\tau _{D}} \biggr)\,dt+\hat{x}^{*} \bigl[U\bigl(1- \hat{u}^{*} \bigr)^{2}-\hat{u}^{*2} \bigr]\frac{dn^{N}(t)}{N}. \end{aligned}$$

Using $A^{N}(t)=\frac{1}{N}\frac{dn^{N}(t)}{dt}$ we recover Eqs. (17a)–(17e) of the main text.

## Appendix 2: Efficient numerical implementation of the mesoscopic approximation

The mesoscopic STP dynamics given by Eqs. (18a)–(18g) are driven by a point process $A^{N}(t)$ or increments $dn(t)$ that are multiplied by a stochastic factor of the form $f(\hat{u}(t),\hat{x}(t))$. In simulations, these stochastic amplitudes require some care. A straightforward discretization of Eqs. (18a)–(18g) would be to draw $\hat{u}(t)$ and $\hat{x}(t)$ from their joint Gaussian distribution (18f) in each time step independently, compute $f(\hat{u}(t),\hat{x}(t))$ and multiply by the number of spikes

46 $$ \Delta n(t)= \int _{t}^{t+\Delta t}dn\bigl(t'\bigr)=N \int _{t}^{t+\Delta t}A\bigl(t'\bigr) \,dt' $$

that occur in the discretization interval $[t,t+\Delta t)$. However, this approach is only correct if the discretization time step Δ*t* is small enough such that Δ*n* contains at most one spike. Because spikes result from a population of many neurons, this condition would require an extremely small time step (such that $Nr(t)\Delta t\ll 1$), and would thus yield a highly inefficient simulation algorithm. Luckily, the independence of the factors $f(\hat{u}(t_{k}),\hat{x}(t_{k}))$ across spikes in the interval $[t,t+\Delta t)$ allows us to use larger time steps that may contain multiple spikes:^1^ due to the independence, the integration of the stochastic term in Eqs. (18a)–(18g) simplifies to a sum of Δ*n**i.i.d.* random variables:

47 $$ \int _{t}^{t+\Delta t}f\bigl(\hat{u}\bigl(t' \bigr),\hat{x}\bigl(t'\bigr)\bigr)\,dn\bigl(t'\bigr)= \sum_{j=1}^{ \Delta n(t)}f(\hat{u}_{j}, \hat{x}_{j}). $$

The $(\hat{u}_{j},\hat{x}_{j})$ are identically distributed because the Gaussian (18f) is held fixed in the time interval $[t,t+\Delta t)$. If the random variables $f(\hat{u}_{j},\hat{x}_{j})$ have mean $\mu _{f}$ and variance $v_{f}$, the sum in Eq. (47) has mean $\mu _{f}\Delta n$ and variance $v_{f}\Delta n$. Using a Gaussian approximation, we can thus approximate the integral, Eq. (47), by

48 $$ \int _{t}^{t+\Delta t}f(\hat{u},\hat{x})\,dn \bigl(t'\bigr)\approx \mu _{f}\Delta n(t)+ \sqrt{ \Delta n(t)}\varepsilon (t), $$

where $\varepsilon (t)$ is a centered Gaussian random variable with variance $v_{f}$ that is independent in each time step.

For example, the integration of the mesoscopic equation for *u*, Eq. (18a), involves the integration of the stochastic term $N^{-1}U(1-\hat{u})\,dn$. Using $\langle \hat{u}\rangle =\mu _{u}=u$ and $\operatorname{var}(\hat{u})=v_{u}=P-u^{2}$, Eq. (48) yields

$$ \frac{U}{N} \int _{t}^{t+\Delta t}\bigl[1-\hat{u} \bigl(t'\bigr)\bigr]\,dn\bigl(t'\bigr)\approx \frac{U}{N} \bigl[ (1-u)\Delta n-\varepsilon _{u}(t)\sqrt{ \Delta n} \bigr], $$

where $\varepsilon _{u}(t)$ is a centered Gaussian random variable with variance $v_{u}$. Similarly, if $(\varepsilon _{u}(t), \varepsilon _{\varepsilon }(t))$ is a pair of correlated centered Gaussian variable with covariance matrix

49 $$ \begin{pmatrix} P(t)-u(t)^{2} & R(t)-u(t)x(t) \\ R(t)-u(t)x(t) & Q(t) - x(t)^{2} \end{pmatrix} , $$

the integration of the mesoscopic equation for *x*, Eq. (18b), yields

50 $$\begin{aligned}& \int _{t}^{t+\Delta t}\hat{u}\bigl(t' \bigr)\hat{x}\bigl(t'\bigr)\,dn\bigl(t'\bigr) \\& \quad = \int _{t}^{t+\Delta t}\bigl(u\bigl(t' \bigr)+\varepsilon _{u}\bigl(t'\bigr)\bigr) \bigl(x \bigl(t'\bigr)+ \varepsilon _{x}\bigl(t' \bigr)\bigr)\,dn\bigl(t'\bigr) \\& \quad = \int _{t}^{t+\Delta t}\bigl[u\bigl(t' \bigr)x\bigl(t'\bigr)+\varepsilon _{u} \bigl(t'\bigr)\varepsilon _{x}\bigl(t' \bigr)\bigr]\,dn\bigl(t'\bigr) \\& \qquad {}+ \int _{t}^{t+\Delta t}\bigl[\varepsilon _{u} \bigl(t'\bigr)x\bigl(t'\bigr)+\varepsilon _{x}\bigl(t'\bigr)u\bigl(t'\bigr) \bigr] \,dn\bigl(t'\bigr) \\& \quad \approx R(t)\Delta n(t)+\bigl[x(t)\varepsilon _{u}(t)+u(t) \varepsilon _{x}(t)\bigr] \sqrt{\Delta n(t)}. \end{aligned}$$

Here, we approximated $\varepsilon _{u}\varepsilon _{x}$ by its mean $\langle \varepsilon _{u}\varepsilon _{x}\rangle =R-ux$, and neglected its fluctuations.

When we integrate Eqs. (18c)–(18e), we encounter the terms $\varepsilon _{u}^{2} \varepsilon _{x}$, $\varepsilon _{u} \varepsilon _{x}^{2}$ and $\varepsilon _{u}^{2} \varepsilon _{x}^{2}$. As we cannot calculate their mean, we perform a moment closure approximation, neglecting all cumulants of order higher than two:

$$ \bigl\langle \varepsilon _{u}^{2} \varepsilon _{x} \bigr\rangle \approx 2 \langle \varepsilon _{u} \rangle \langle \varepsilon _{u} \varepsilon _{x}\rangle + \bigl\langle \varepsilon _{u}^{2} \bigr\rangle \langle \varepsilon _{x} \rangle -2\langle \varepsilon _{u} \rangle ^{2} \langle \varepsilon _{x}\rangle = 0; $$

symmetrically, $\langle \varepsilon _{u} \varepsilon _{x}^{2} \rangle \approx 0$; and finally,

$$\begin{aligned} \bigl\langle \varepsilon _{u}^{2} \varepsilon _{x}^{2} \bigr\rangle &\approx \bigl\langle \varepsilon _{u}^{2} \bigr\rangle \bigl\langle \varepsilon _{x}^{2} \bigr\rangle + 2\langle \varepsilon _{u} \varepsilon _{x} \rangle ^{2} \\ &= \bigl(P-u^{2}\bigr) \bigl(Q-x^{2}\bigr) + 2(R-ux)^{2}. \end{aligned}$$

These approximations allow for the completion of the derivation.

In summary, the Euler scheme corresponding to Eqs. (18a)–(18g) is

$$\begin{aligned}& u(t+\Delta t)=u(t)+\Delta u, x(t+\Delta t)=x(t)+\Delta x, \\& P(t+\Delta t)=P(t)+\Delta P, Q(t+\Delta t)=Q(t)+\Delta Q, \\& R(t+\Delta t)=R(t)+\Delta R, \end{aligned}$$

with increments given by

51a $$\begin{aligned}& \Delta u =\frac{U_{0}-u}{\tau _{F}}\Delta t+\frac{U}{N} \bigl[(1-u) \Delta n - \varepsilon _{u}\sqrt{\Delta n} \bigr], \end{aligned}$$

51b $$\begin{aligned}& \Delta x =\frac{1-x}{\tau _{D}}\Delta t-\frac{1}{N} \bigl[R\Delta n +(u \varepsilon _{x} + x\varepsilon _{u})\sqrt{\Delta n} \bigr], \end{aligned}$$

51c $$\begin{aligned}& \Delta P =2 \frac{U_{0}u-P}{\tau _{F}}\Delta t+\frac{1}{N} [ \mu _{P}\Delta n +\varepsilon _{P}\sqrt{\Delta n} ], \end{aligned}$$

51d $$\begin{aligned}& \Delta Q =2 \frac{x-Q}{\tau _{D}}\Delta t+\frac{1}{N} [ \mu _{Q}\Delta n +\varepsilon _{Q}\sqrt{\Delta n} ], \end{aligned}$$

51e $$\begin{aligned}& \Delta R = \frac{U_{0}x-R}{\tau _{F}}\Delta t+ \frac{u-R}{\tau _{D}}\Delta t+ \frac{1}{N} [ \mu _{R}\Delta n +\varepsilon _{R}\sqrt{\Delta n} ]. \end{aligned}$$

Here, we abbreviated

$$\begin{aligned}& \mu _{P} = U \bigl(P (U - 2) - 2 u (U - 1) + U \bigr), \\& \varepsilon _{P} = 2U \bigl(1 + u (U - 2) - U \bigr)\varepsilon _{u}, \\& \mu _{Q} = P Q - 2 Q u + 2 \bigl(R + (u - 2) x \bigr) (R - u x), \\& \varepsilon _{Q} = 2(u - 1)x^{2}\varepsilon _{u} + 2u(u-2)x \varepsilon _{x}, \\& \begin{aligned} \mu _{R} &= \bigl(U (1 - u)^{2} - u^{2} \bigr) x + (U - 1) x \bigl(P - u^{2}\bigr) \\ &\quad {} + 2 \bigl(U (u - 1) - u\bigr) (R - u x), \end{aligned} \\& \varepsilon _{R} = 2 \bigl(U(u - 1) - u \bigr)x\varepsilon _{u} + \bigl(U(1 - u)^{2} - u^{2} \bigr) \varepsilon _{x}. \end{aligned}$$

The generation of correlated centered Gaussian random variables $\varepsilon _{u}(t)$ and $\varepsilon _{x}(t)$ with covariance matrix (49) can be implemented by standard methods. For instance, one may compute in each time step the correlation coefficient

52 $$ \rho =\frac{R-ux}{\sqrt{(P-u^{2})(Q-x^{2})}}. $$

In the initial warm-up phase, it may happen that the numerical values of the variances $P-u^{2}$ and $Q-x^{2}$ are non-positive or the absolute value of the correlation coefficient $|\rho |$ exceeds unity. One simple practical solution to this problem is to set $\varepsilon _{u}$ and $\varepsilon _{x}$ to zero in these cases. Otherwise, we generate random variables by the formula

53 $$\begin{aligned}& \varepsilon _{u} =\sqrt{P-u^{2}}z_{1}, \end{aligned}$$

54 $$\begin{aligned}& \varepsilon _{x} =\sqrt{Q-x^{2}} \bigl( \rho z_{1}+\sqrt{1-\rho ^{2}}z_{2} \bigr), \end{aligned}$$

where $z_{1}$ and $z_{2}$ are independent standard normal random numbers.

Finally, we note that in numerical simulations it is convenient to operate on the spike counts $\Delta n(t)$ rather than on the population activity $A^{N}(t)$. The discretized population activity can be easily obtained from the spike counts via the formula

55 $$ A^{N}(t)=\frac{\Delta n(t)}{N\Delta t}. $$

## Appendix 3: Equations for infinite-size populations and steady-state formulas

A macroscopic theory of STP for infinite-size populations for what we call the first-order MF has been presented in [30]. We detail here the adaptation of our second-order MF to the case if infinite-size populations.

In the infinite-size case, the stochastic populations activity $A^{N}(t)$ becomes a deterministic rate $r(t)$, which simplifies our mesoscopic equations (18a)–(18g): in the stochastic ODEs, $r(t)$—being deterministic—is not multiplied by a random term but by the mean of this term, transforming the stochastic ODEs into deterministic ODEs. These means have already been computed in Appendix B. Hence, in the infinite-size case, our second-order MF equations are

56a $$\begin{aligned}& \frac{\mathrm{d}u}{\mathrm{d}t} =\frac{U_{0}-u}{\tau _{F}}+U(1 - u)r(t), \end{aligned}$$

56b $$\begin{aligned}& \frac{\mathrm{d}x}{\mathrm{d}t} =\frac{1-x}{\tau _{D}}-R r(t), \end{aligned}$$

56c $$\begin{aligned}& \frac{\mathrm{d}P}{\mathrm{d}t} =2 \frac{U_{0}u-P}{\tau _{F}}+ \mu _{P} r(t), \end{aligned}$$

56d $$\begin{aligned}& \frac{\mathrm{d}Q}{\mathrm{d}t} =2\frac{x-Q}{\tau _{D}}+ \mu _{Q} r(t), \end{aligned}$$

56e $$\begin{aligned}& \frac{\mathrm{d}R}{\mathrm{d}t} = \frac{U_{0}x-R}{\tau _{F}}+\frac{u-R}{\tau _{D}}+ \mu _{R} r(t). \end{aligned}$$

Again, we abbreviated

$$\begin{aligned}& \mu _{P} = U \bigl(P (U - 2) - 2 u (U - 1) + U \bigr), \\& \mu _{Q} = P Q - 2 Q u + 2 \bigl(R + (u - 2) x \bigr) (R - u x), \\& \begin{aligned} \mu _{R} &= \bigl(U (1 - u)^{2} - u^{2} \bigr) x + (U - 1) x \bigl(P - u^{2}\bigr) \\ &\quad {} + 2 \bigl(U (u - 1) - u\bigr) (R - u x). \end{aligned} \end{aligned}$$

Given a fixed rate *r*, we can compute the steady-state values of *u*, *x*, *P*, *Q* and *R*:

57a $$\begin{aligned}& u = \frac{\tau _{F} r U+U_{0}}{\tau _{F} r U+1}, \end{aligned}$$

57b $$\begin{aligned}& P = \frac{\tau _{F} r U (2 u (U-1)-U)-2 u U_{0}}{\tau _{F} r (U-2) U - 2}, \end{aligned}$$

57c $$\begin{aligned}& x = \frac{\tau _{D} \tau _{F} r (-2 u U+u+2 U)+\tau _{D}+\tau _{F}}{Z}, \end{aligned}$$

57d $$\begin{aligned}& R = \frac{\tau _{D} (\tau _{F} r (P (U-1)-2 u^{2} (U-1)+U )+U_{0} )+\tau _{F} u}{Z}, \end{aligned}$$

57e $$\begin{aligned}& Q = \frac{-2 \tau _{D} r (R+(u-2) x) (R-u x)-2 x}{\tau _{D} r (P-2 u)-2}. \end{aligned}$$

We used the abbreviation

$$\begin{aligned} Z &= \tau _{D}^{2} r \bigl(\tau _{F} r \bigl(P (U-1)-2 u^{2} (U-1)+U \bigr)+U_{0} \bigr) \\ &\quad {} +2 \tau _{D} \tau _{F} r (-u U+u+U)+\tau _{D}+\tau _{F}. \end{aligned}$$

Note that these steady-states formulas are very useful for carrying out precise phase-plane analyses.

## Appendix 4: Mesoscopic population equations for network of GIF neurons—ODE representation

The equation for the population rate $r(t)$ for GIF neurons involves the integral, Eq. (31), over all possible refractory states and a set of partial differential equations (so-called quasi-linear equations) for the quantities $q(\tau ,t)$, $V(\tau ,t)$ and $W(\tau ,t)$. Instead of the age of the neuron (i.e. the time *since* its last spike), the refractory state can be equivalently specified by the last spike time $\hat {t}=t-\tau $. This variable transformation turns the partial differential equations into ordinary differential equations (ODEs), which yields an alternative formulation of the mesoscopic population equations.

If the refractory state is specified by the last spike time $\hat {t}=t-\tau $, we need to consider the density of last spike times $Q(\hat {t},t)\equiv q(t-\hat {t},t)$. Instead of *Q*, it is slightly more convenient to write $Q(\hat {t},t)=S(t|\hat {t})A^{N}(\hat {t})$, where we introduced the survivor function $S(t|\hat {t})$. With this notation the population rate, Eq. (31), becomes [12]

58 $$ r(t)= \int _{-\infty }^{t}\lambda (t|\hat {t})S(t|\hat {t})A^{N}(\hat {t}) \,d\hat {t}+ \varLambda (t) \biggl( 1- \int _{-\infty }^{t}S(t|\hat {t})A^{N}(\hat {t}) \,d\hat {t}\biggr). $$

Using the method of characteristics, the quasi-linear equation (32) for $q(\tau ,t)$ has an equivalent ODE representation for the characteristic curves $\{Q(\hat {t},t)\}_{t>\hat {t}}$: $dQ/dt=-\lambda Q$ with initial condition $Q(\hat {t},\hat {t})=A^{N}(\hat {t})$. This corresponds to an ODE for the survivor function:

59 $$ \frac{\mathrm{d}S(t|\hat {t})}{\mathrm{d}t}=-\lambda (t|\hat {t})S(t|\hat {t}),\qquad S(\hat {t}|\hat {t})=1. $$

The functions $\lambda (t|\hat {t})\equiv \lambda (t-\hat {t},t)$ and $\varLambda (t)$ follow from Eq. (33) as

60 $$ \lambda (t|\hat {t})=c\exp \biggl( \frac{V(t|\hat {t})- \vartheta (t|\hat {t})}{\Delta _{u}} \biggr),\qquad \varLambda (t)= \frac{\int _{-\infty }^{t}\lambda (t|\hat {t})W(t|\hat {t}) \, \mathrm{d}\hat {t}}{\int _{-\infty }^{t}W(t|\hat {t})\, \mathrm{d}\hat {t}}. $$

The dynamics for the variables $V(t|\hat {t})$ and $W(t|\hat {t})$ follow from those of the quasi-linear equations (34) and (35):

61 $$\begin{aligned}& \frac{\mathrm{d}V(t|\hat {t})}{\mathrm{d}t} =- \frac{V(t|\hat {t}) -\mu }{\tau _{\mathrm {m}}}+ \frac{R_{\mathrm{m}}}{\tau _{\mathrm {m}}} I_{ \mathrm{syn}}(t), \end{aligned}$$

62 $$\begin{aligned}& \frac{\mathrm{d}W(t|\hat {t})}{\mathrm{d}t} =-\lambda (t|\hat {t})\bigl[2W(t| \hat {t}) -S(t|\hat {t})A^{N}(\hat {t}) \bigr], \end{aligned}$$

with initial conditions $V(\hat {t}|\hat {t})=V_{\mathrm {r}}$ and $W(\hat {t}|\hat {t})=0$. Finally, the threshold function $\vartheta (t|\hat {t})$ reads

63 $$ \vartheta (t|\hat {t})=V_{\mathrm {th}}+\theta (t-\hat {t})+ \int _{-\infty }^{\hat {t}} \tilde{\theta } \bigl(t-t'\bigr)A^{N}\bigl(t'\bigr) \, \mathrm{d}t'. $$

## Appendix 5: Recurrent network parameters

Parameters of Fig. 6, Fig. 7 and Fig. 8 are detailed in Table 1.

**Table 1:** Parameters of Fig. 6, Fig. 7 and Fig. 8

Parameter	Unit	Fig. 6	Fig. 7	Fig. 8
$\tau _{D}$	s	0.2	0.3	0.05
$\tau _{F}$	s	0.2	0.3	0.002
*U*		0.2	0.2	0.6
$U_{0}$		0.2	0.2	0.6
$\tau _{\mathrm {m}}$	s	0.02	0.02	0.02
$\tau _{s}$	s	0.002	0.002	0.01
*μ*	mV	−13.3	−13.1	−8.7
$R_{\text{m}}$	Ω	1	1	1
*β*		0.4	0.4	0.5
$r_{\text{max}}$	Hz	500	500	200
$h_{0}$	mV	0	0	0
*J*⋅*N*⋅*p*		78.5	83.5	28.0

In Fig. 9, we used the following parameters: $\tau _{D} = 0.5\mbox{ s}$, $\tau _{F} = 0.01\mbox{ s}$, $U = 0.1$, $U_{0} = 0.1$, $\tau _{\mathrm {m}}= 0.015\mbox{ s}$, $\tau _{s} = 0.01\mbox{ s}$, $c = 15\text{ Hz}$, $V_{\mathrm{reset}} = 0\mbox{ mV}$, $V_{\mathrm{th}} = 15\mbox{ mV}$, $\mu = 4\mbox{ mV}$, $R_{\mathrm{m}} = 1~\Omega$, $\Delta _{u} = 5\mbox{ mV}$, $t_{\mathrm{ref}} = 0.004\mbox{ s}$, $J\cdot N = 850$.
